# Supplementary material for: Multiple cancer pathways regulate telomere protection
Source: EMBO Mol Med. 2019 Jun 13;11(7):e10292. doi: 10.15252/emmm.201910292 (PMC6609915; doi:10.15252/emmm.201910292)
Supplement: Supplementary file 1 — Appendix [file EMMM-11-e10292-s001.pdf]

# Multiple cancer pathways regulate telomere protection

Leire Bejarano[1,#], Giuseppe Bosso[1,#], Jessica Louzame[1], Rosa Serrano[1], Elena Gómez-Casero[2], Jorge Martínez-Torrecuadrada[3], Sonia Martínez[2], Carmen Blanco-Aparicio[2], Joaquín Pastor[2] and Maria A. Blasco[1*]

## APPENDIX

- **Appendix figure legends**

- ***Appendix Figure S1*: Effect of TRF1 inhibitory compounds on RAP1 and TIN2 protein levels.**

- ***Appendix Figure S2*.:TRF1 inhibitors induce telomere induced foci in lung cancer cells.**

- ***Appendix Figure S3:* Novel TRF1 inhibitors can act independently of the PI3K-AKT pathway.**

- ***Appendix Figure S4*: Cell numbers upon transduction with eGFP-*Trf1* WT or the indicated mutant alleles at day 8**

- ***Appendix Figure S5: In vivo* combinatorial studies with the new TRF1 inhibitory compounds using patient-derived GBM xenograft models**

- ***Appendix Table S1*: Chemical compounds tested in a primary screening for identifying novel compounds with the ability to downregulate TRF1 protein levels**

- **Appendix Table S2: P-values**

**Appendix Figure S1. Effect of TRF1 inhibitory compounds on RAP1 and TIN2 protein levels. (A)** TIN2 Western blot images (left) and quantification of TIN2 protein levels (right) of CHA9-3 lung cancer cells treated with the indicated compounds for 24h at 1 μM. Data are representative of n=8 (DMSO) and n=4 (HSP90i, Aurorai, Docetaxel, PLKi, CDKi, mTORi, Gemcitabine, MEKi, ERKi, RTKi) independent experiments. **(B)** RAP1 Western blot images (left) and quantification of RAP1 protein levels (right) of CHA9-3 lung cancer cells treated with the indicated compounds for 24h at 1 μM. Data are representative of n=8 (DMSO) and n=4 (HSP90i, Aurorai, Docetaxel, PLKi, CDKi, mTORi, Gemcitabine, MEKi, ERKi, RTKi) independent experiments. Data are represented as mean ± SEM. Significant differences using unpaired *t*-test are indicated by $*P < 0.05$, $**P < 0.01$, $***P < 0.001$.

**Appendix Figure S2. TRF1 inhibitors induce telomere induced foci in lung cancer cells. (A)** Quantification of γH2AX nuclear intensity upon treatment of CHA9-3 lung cancer cells with the indicated compounds. Data are representative of n=6 (DMSO) and n=3 (PI3Ki, mTORi, RTKi, ERKi, MEKi, HSP90i, Docetaxel, CDKi) independent experiments. **(B)** Quantification of mean number of telomere induced foci (TIF) per nucleus in CHA9-3 lung cancer cells treated with the indicated compounds for 24h at 1 μM. Data are representative of n=3 independent experiments. **(C)** Mean number of TIFs per nucleus in CHA9.3 lung cancer cells normalized to γH2AX nuclear intensity. Data are representative of n=3 independent experiments. Data are represented as mean ± SEM. Significant differences using unpaired *t*-test are indicated by $^{*}P < 0.05$, $^{**}P < 0.01$, $^{***}P < 0.001$.

**Appendix Figure S3. Novel TRF1 inhibitors can act independently of the PI3K-AKT pathway. (A)** Western blot images (up) and p-AKT/AKT protein levels (down) of h676 GSCs treated with the indicated compounds for 24h at 1 μM. Data are representative of n=2 independent experiments. Data are represented as mean ± SEM. Significant differences using unpaired *t*-test are indicated by *$P$ < 0.05, **$P$ < 0.01.

**Appendix Figure S4**. Cell numbers upon transduction with eGFP-*Trf1* WT or the indicated mutant alleles at day 8. Statistical significance is indicated in the table (*) between the different conditions (down). Data are representative of n=5 independent experiments. Data are represented as mean ± SEM. Significant differences using ANOVA test Bonferroni are indicated by *$P$ < 0.05, **$P$ < 0.01.

**Appendix Figure S5.** ***In vivo*** **combinatorial studies with the new TRF1 inhibitory compounds using patient-derived GBM xenograft models. (A)** Quantification of the percentage of mice died during treatment with the indicated compounds as single agents or in combination. n represents number of mice: vehicle n=8; PI3K n=17; MEKi, Docetaxel, Gemcitabine, RTKi, ERKi and their combinations with PI3Ki n=4; HSP90i and the combination with PI3K n=2. Significant differences using Chi-Square are indicated by $*P < 0.05$, $**P < 0.01$, $***P < 0.001$. **(B-C)** Longitudinal tumor growth follow-up in mice injected with h676 GSCs and treated with the indicated compounds as single agents or combination. Data are represented as mean ± SEM. n represents number of tumors: in B: vehicle n=16, PI3Ki n=16, RTKi n=8, Combination n=8; in C: vehicle n=16, PI3Ki n=16, HSP90i n=4, Combination n=4 . Significant differences using unpaired *t*-test are indicated by $*P < 0.05$, $**P < 0.01$, $***P < 0.001$.

Appendiix Figure S1

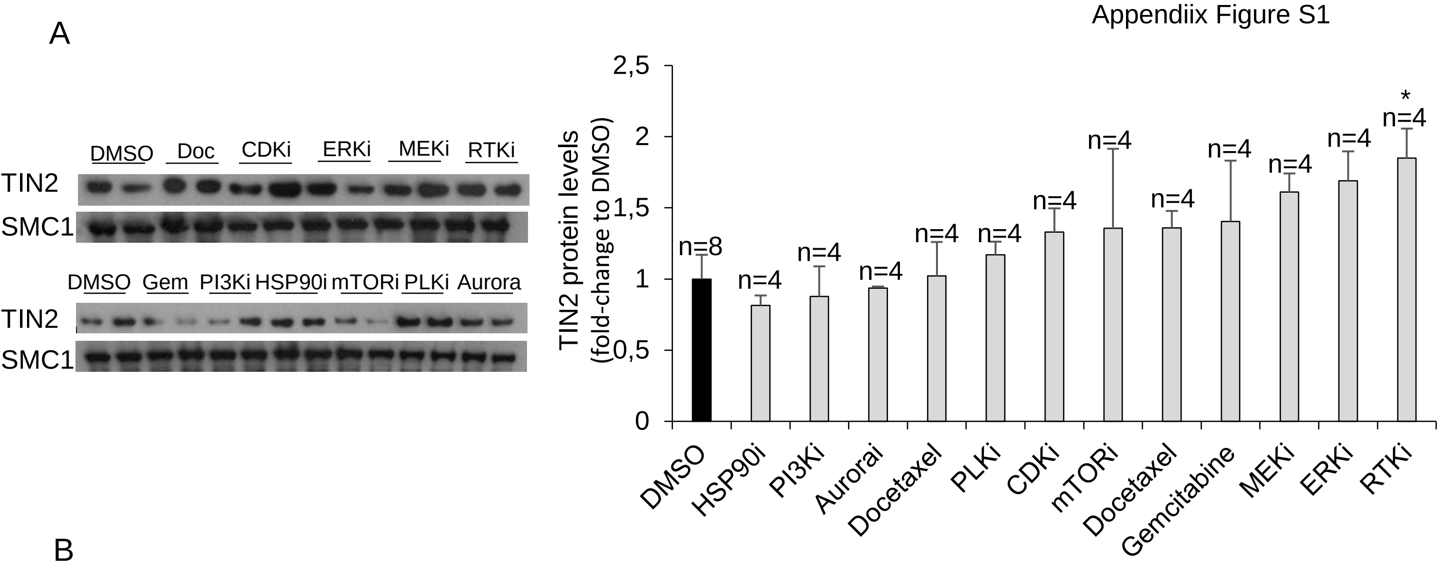


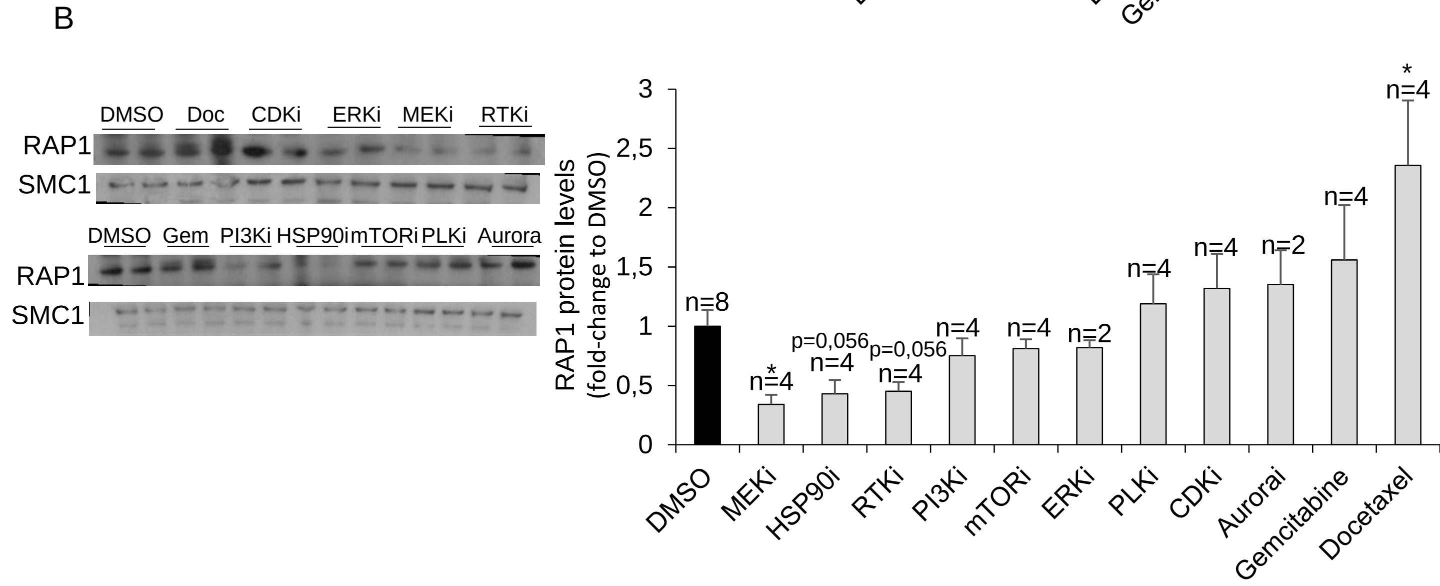

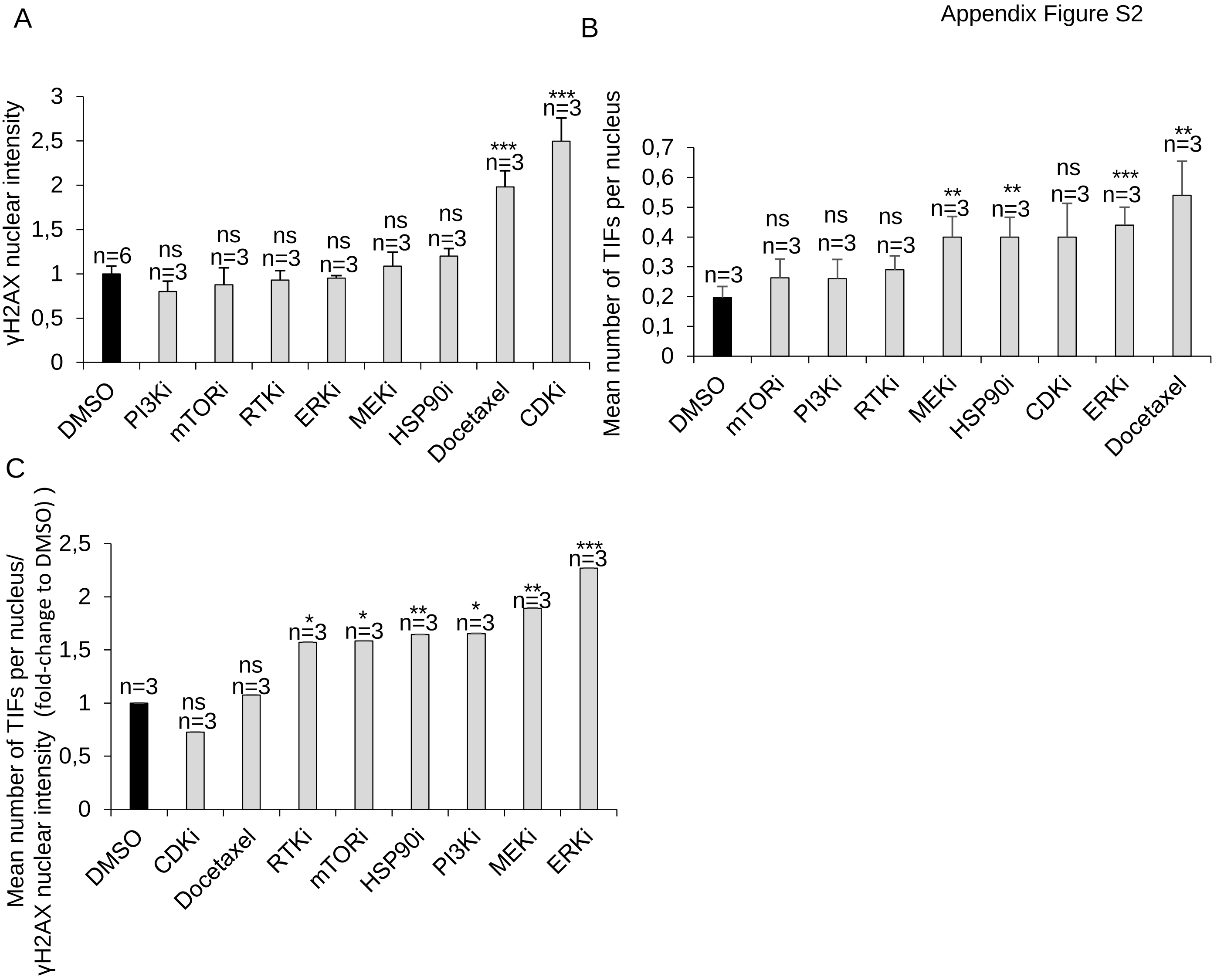
A
γH2AX nuclear intensity
3
2,5
2
1,5
1
0,5
0
n=6
ns n=3
ns n=3
ns n=3
ns n=3
ns n=3
ns n=3
*** n=3
*** n=3
DMSO
PI3Ki
mTORi
RTKi
ERKi
MEKi
HSP90i
Docetaxel
CDKi
B
Mean number of TIFs per nucleus
0,7
0,6
0,5
0,4
0,3
0,2
0,1
0
n=3
ns n=3
ns n=3
ns n=3
** n=3
** n=3
ns n=3
*** n=3
** n=3
DMSO
mTORi
PI3Ki
RTKi
MEKi
HSP90i
CDKi
ERKi
Docetaxel
C
Mean number of TIFs per nucleus/
γH2AX nuclear intensity (fold-change to DMSO) )
2,5
2
1,5
1
0,5
0
n=3
ns n=3
ns n=3
* n=3
* n=3
** n=3
* n=3
** n=3
*** n=3
DMSO
CDKi
Docetaxel
RTKi
mTORi
HSP90i
PI3Ki
MEKi
ERKi

A

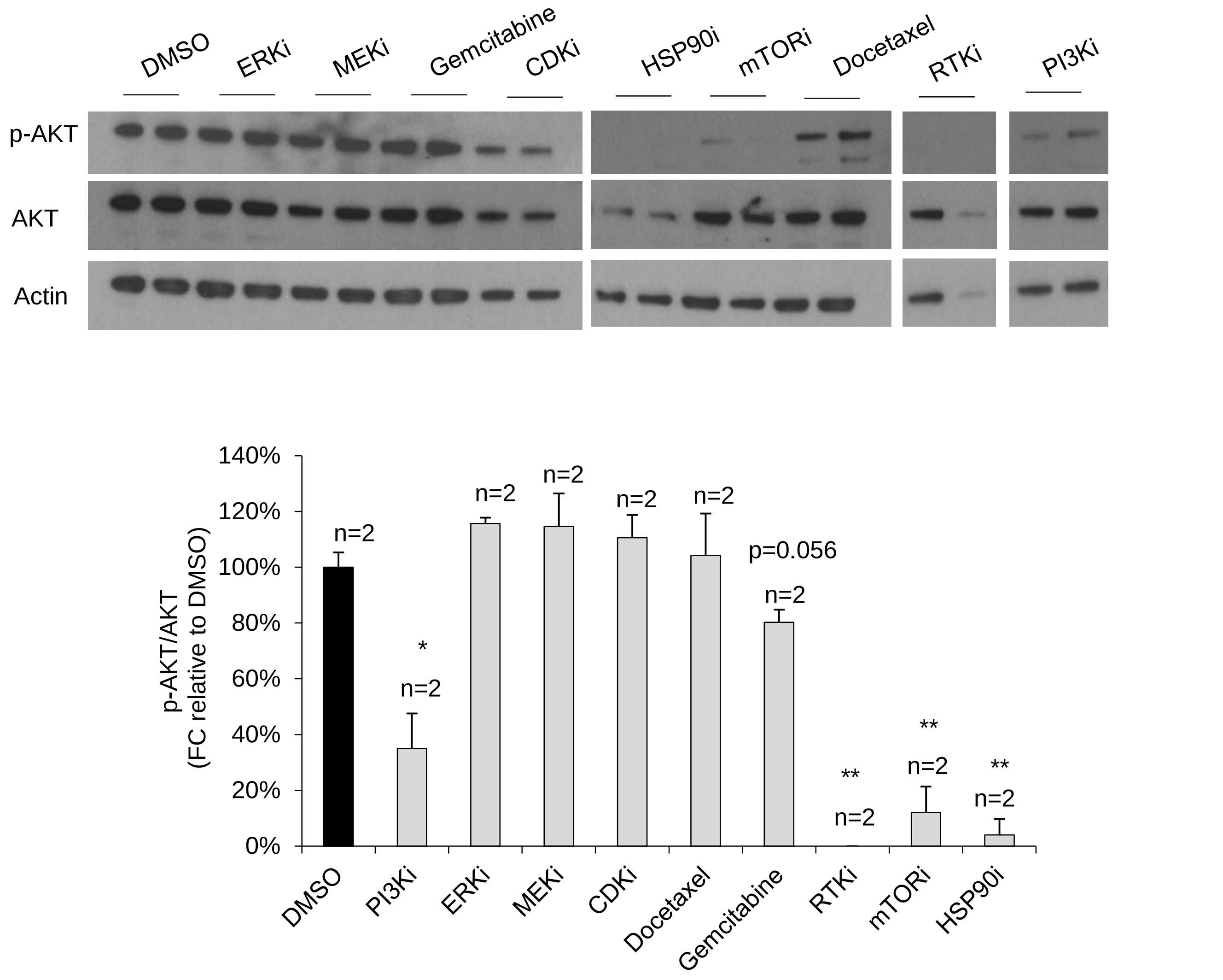
DMSO
ERKi
MEKi
Gemcitabine
CDKi
HSP90i
mTORi
Docetaxel
RTKi
PI3Ki
p-AKT
AKT
Actin
140%
120%
100%
80%
60%
40%
20%
0%
p-AKT/AKT
(FC relative to DMSO)
n=2
*
p=0.056
**
DMSO
PI3Ki
ERKi
MEKi
CDKi
Docetaxel
Gemcitabine
RTKi
mTORi
HSP90i

Appendix Figure S4

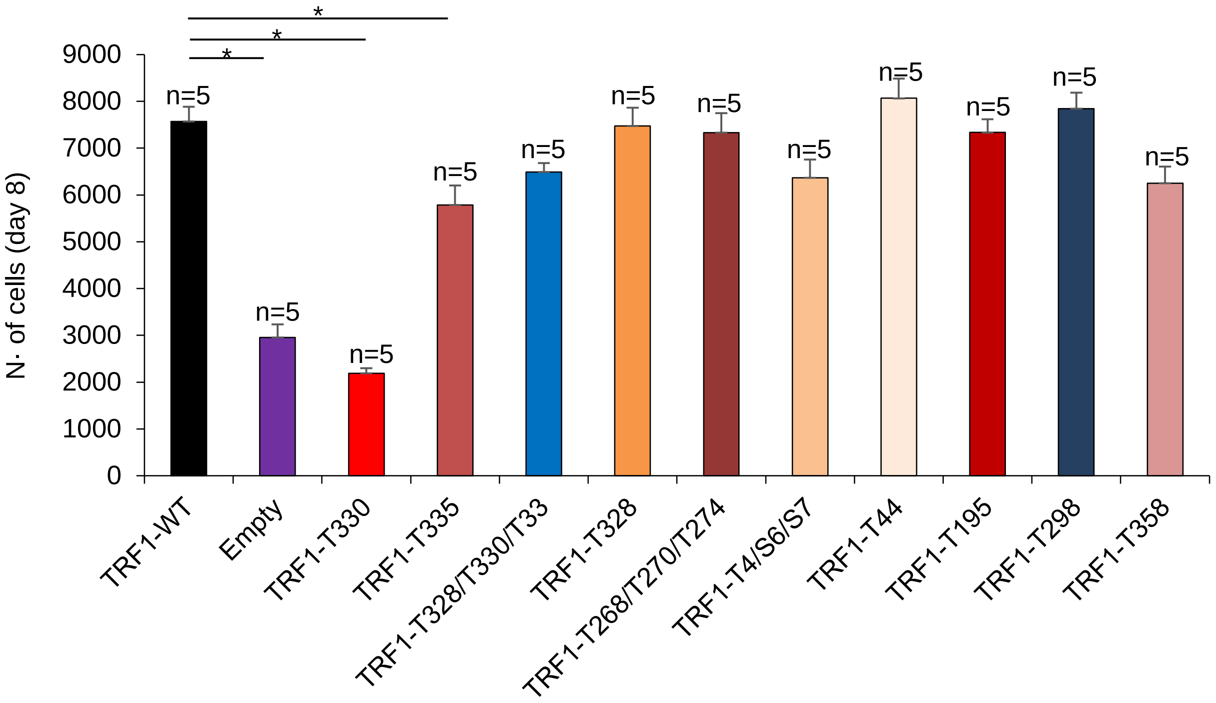


ANOVA test - Bonferroni

| | Cre | WT | T44 | T195 | T298 | T328 | T330 | T335 | T4/S6/S7 | T358 | T268/T270/T274 | T328/T330/T33 |
|---|---|---|---|---|---|---|---|---|---|---|---|---|
| Cre | | * | * | * | * | * | | * | * | * | * | * |
| WT | * | | | | | | * | * | | | | |
| T44 | * | | | | | | * | * | | * | | |
| T195 | * | | | | | | * | | | | | |
| T298 | * | | | | | | * | * | | | | |
| T328 | * | | | | | | * | | | | | |
| T330 | | * | * | * | * | * | | * | * | * | * | * |
| T335 | * | * | * | | * | | * | | | | | |
| T4/S6/S7 | * | | | | | | * | | | | | |
| T358 | * | | * | | | | * | | | | | |
| T268/T270/T274 | * | | | | | | * | | | | | |
| T328/T330/T33 | * | | | | | | * | | | | | |

*The mean difference is significance at the 0,05 level

Appendix Figure S5

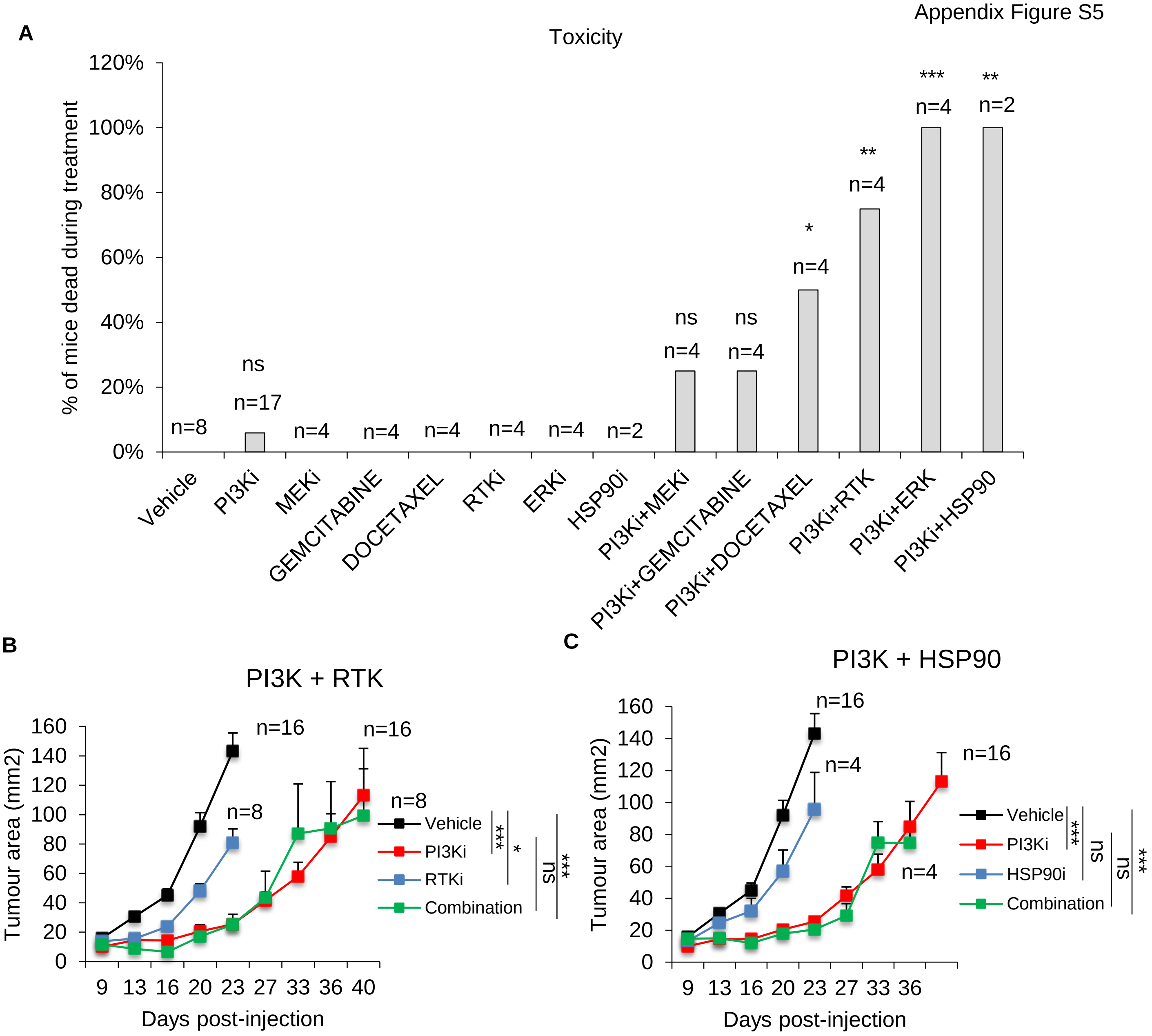

**Appendix table S1.** Chemical compounds tested in a primary screening for identify novel compounds with the ability to downregulate TRF1 protein levels.
A cut-off above 20% inhibition of TRF1 intensity was established to consider a drug as a hit. Color code indicate those drugs that inhibit the same target. A retest in one representative of each class was done to confirm the hits and consider true hits those with an inhibition or TRF1 levels above 20%.

| **drug_name** | Inhibition sum_int2_area Vs DMSO (%) | sd | **Retest by confocal** | Inhibition sum_int2_area Vs DMSO (%) in validation assay |
|---|---|---|---|---|
| GEDATOLISIB | 41,765 | 0,54 | | |
| BEZ-235 | 40,515 | 6,16 | | |
| GSK2126458 | 39,43 | 4,62 | | |
| CNIO-PI3K-3 | 40,785 | 2,67 | | |
| GDC-0941 | 39,5 | 0,03 | | |
| ZSTK474 | 37,77 | 0,30 | Yes | 38,9 |
| BKM120 | 35,69 | 2,47 | | |
| PI3K-CNIO-1 | 32,975 | 3,75 | | |
| CNIO-PI3K-2 | 25 | 7,07 | | |
| KU-0063794 | 35,24 | 0,96 | | |
| RAPAMYCIN | 28,275 | 5,10 | | |
| MK-2206 | 29,585 | 0,15 | | |
| GSK461364 | 38,135 | 0,64 | Yes | 26,38 |
| SELUMETINIB | 34,105 | 0,33 | Yes | 21,55 |
| TRAMETINIB | 32,615 | 0,80 | | |
| PD-0325901 | 28,07 | 0,35 | | |
| SCH772984 | 38,89 | 0,37 | Yes | 35,58 |
| DASATINIB | 32,895 | 2,27 | yes | 42,85 |
| TAXOTERE | 32,03 | 1,53 | Yes | 26,94 |
| VINCRISTINE | 23,925 | 1,61 | | |
| PACLITAXEL | 27,375 | 0,04 | | |
| CNIO-ATR-1 | 30,445 | 1,36 | Yes | <5 |
| AZ20 | 22,99 | 1,94 | | |
| GELDANAMYCIN | 30,255 | 0,05 | Yes | 45,43 |
| PANOBINOSTAT | 23,745 | 0,19 | Yes | <5 |
| 5-Fluoracil | 21,965 | 1,03 | Yes | <5 |
| ALISERTIB | 21,57 | 2,94 | Yes | 26,11 |
| VX-680 | 21,395 | 2,26 | | |
| GEMCITABINE | 21,115 | 1,80 | Yes | 38,03 |
| CRIZOTINIB | 20,125 | 2,37 | Yes | 15,29 |
| FLAVOPIRIDOL | 46,66 | 0,38 | Yes | 86,34 |
| DOVITINIB | 19,915 | 0,86 | | |
| BYL-719 | 19,735 | 11,29 | | |
| CUDC-907 | 18,39 | 1,85 | | |
| Mitomycin C | 17,85 | 0,27 | | |
| SAHA | 17,74 | 1,88 | | |
| CUDC-101 | 17,635 | 3,10 | | |
| PERIFOSINE | 16,84 | 1,57 | | |
| SNS-314 MESYLATE | 16,54 | 1,05 | | |
| Ixazomib | 14,805 | 1,96 | | |
| KU-57788 | 14,165 | 4,41 | | |
| DABRAFENIB | 13,73 | 3,45 | | |
| CARFILZOMIB | 13,31 | 3,35 | | |
| PEMETREXED | 13,255 | 2,13 | | |
| ETOPOSIDE | 12,99 | 0,92 | | |
| GDC-0994 | 12,24 | 2,46 | | |
| BORTEZOMIB | 11,06 | 1,51 | | |
| TX-1123 | 10,91 | 5,61 | | |
| ABIRATERONE | 8,79 | 5,43 | | |
| AT7519 | 8,61 | 2,73 | | |
| AICAR | 7,79 | 10,49 | | |
| TEMOZOLOMIDE | 7,05 | 13,31 | | |
| SN-38 | 5,845 | 0,23 | | |
| OLAPARIB | 5,075 | 5,35 | | |
| ELESCLOMOL | 5,02 | 3,44 | | |
| BARDOXOLONE METHYL | < 5 | 2,75 | | |
| BAY 61-3606 | < 5 | 4,78 | | |
| BAY 87-2243 | < 5 | 9,50 | | |
| BIRB 796 | < 5 | 6,52 | | |
| CISPLATINUM | < 5 | 4,21 | | |
| CNIO-PIM-1 | < 5 | 6,92 | | |
| CYCLOPHOSPHAMIDE | < 5 | 2,57 | | |
| Dibenzazepine | < 5 | 2,81 | | |

**Appendix table S1.** Chemical compounds tested in a primary screening for identify novel compounds with the ability to downregulate TRF1 protein levels.
A cut-off above 20% inhibition of TRF1 intensity was established to consider a drug as a hit. Color code indicate those drugs that inhibit the same target. A retest in one representative of each class was done to confirm the hits and consider true hits those with an inhibition or TRF1 levels above 20%.

| **drug_name** | Inhibition sum_int2_area Vs DMSO (%) | sd | **Retest by confocal** | Inhibition sum_int2_area Vs DMSO (%) in validation assay |
|---|---|---|---|---|
| EFLORNITHINE | < 5 | 11,05 | | |
| ERLOTINIB | < 5 | 55,30 | | |
| EX-527 | < 5 | 6,77 | | |
| Fulvestrant | < 5 | 6,12 | | |
| Galunisertib | < 5 | 1,53 | | |
| GEFITINIB | < 5 | 7,88 | | |
| GENISTEIN | < 5 | 26,00 | | |
| GLEEVEC, IMATINIB | < 5 | 10,94 | | |
| IRINOTECAN | < 5 | 2,25 | | |
| KETOCONAZOLE | < 5 | 0,11 | | |
| LAPATINIB | < 5 | 3,42 | | |
| LETROZOLE | < 5 | 0,83 | | |
| LINIFANIB | < 5 | 2,57 | | |
| LOMUSTINE | < 5 | 25,63 | | |
| LY2801653 | < 5 | 29,07 | | |
| METFORMIN | < 5 | 9,60 | | |
| Mifepristone | < 5 | 7,45 | | |
| MK-906 | < 5 | 10,44 | | |
| OSI-906 | < 5 | 10,97 | | |
| OXALIPLATIN | < 5 | 44,07 | | |
| Palbociclib | < 5 | 10,76 | | |
| Pazopanib | < 5 | 47,46 | | |
| 4-PB (SODIUM 4-PHENYLBUTYRATE) | < 5 | 2,45 | | |
| PF 4708671 | < 5 | 27,01 | | |
| PFK15 | < 5 | 5,18 | | |
| PX-478 | < 5 | 3,81 | | |
| QUIZARTINIB | < 5 | 0,68 | | |
| Roscovitine | < 5 | 7,42 | | |
| SB 203580 | < 5 | 19,71 | | |
| SB 505124 | < 5 | 10,03 | | |
| SEMAGACESTAT | < 5 | 50,28 | | |
| Silmitasertib | < 5 | 18,57 | | |
| SORAFENIB | < 5 | 40,46 | | |
| S-RUXOLITINIB | < 5 | 1,93 | | |
| Suramin | < 5 | 5,13 | | |
| Tamoxifen | < 5 | 3,52 | | |
| TANZISERTIB | < 5 | 27,20 | | |
| Tempol | < 5 | 33,45 | | |
| VALPROIC ACID | < 5 | 9,46 | | |
| Vemurafenib | < 5 | 25,14 | | |
| VISMODEGIB | < 5 | 15,05 | | |
| Zileuton | < 5 | 18,31 | | |
| AZD5363 | <5 | 18,07 | | |
| CAL-101 | <5 | 16,74 | | |
| DISULFIRAM | <5 | 0,74 | | |
| GSK2636771 | <5 | 10,34 | | |
| PILARALISIB | <5 | 18,58 | | |
| TGX-221 | <5 | 24,34 | | |
| NVP-BGJ398 | >5 | 11,62 | | |
| Ricolinostat | >5 | 0,05 | | |

# Appendix Table S2: P Values

| FIGURE | SAMPLE | P VALUE |
|---|---|---|
| Figure 1C | | |
| DMSO | Aurorai | 0,0503 |
| | HSP90i | 0,0041 |
| | mTORi | 0,0054 |
| | Docetaxel | 0,0116 |
| | ERKi | 1,77E-05 |
| | MEKi | 2,08E-05 |
| | Gemcitabine | 0,0007 |
| | CDKi | 0,0021 |
| | RTKi | 6,50E-05 |
| | PLKi | 0,1330 |
| Figure 2A | | |
| DMSO | ERKi | 0,0014 |
| | MEKi | 0,0169 |
| | RTKi | 0,0420 |
| | HSP90i | 0,0004 |
| | CDKi | 0,0054 |
| | mTORi | 0,2388 |
| | PI3Ki | 0,0118 |
| | Docetaxel | 0,0045 |
| Figure 2B | Sample | PVALUE |
| DMSO | mTORi | 0,0371 |
| | Docetaxel | 0,0207 |
| | ERKi | 0,0371 |
| | MEKi | 0,0199 |
| | RTKi | 0,0133 |
| | HSP90i | 0,0028 |
| | Gemcitabine | 0,0079 |
| | CDKi | 0,0003 |
| Figure 2C | | |
| DMSO | mTORi | 2,87E-05 |
| | MEKi | 0,0033 |
| | RTKi | 1,58E-08 |
| Figure 5C | Sample | PVALUE |
| TRF1-WT | T44 | 0,4232 |
| | T195 | 0,9891 |
| | T298 | 0,2465 |
| | T358 | 0,7300 |
| | T4 S6 S7 | 0,1457 |
| | T268 T270 T274 | 0,5850 |
| | T328 T330 T335 | 3,68E-05 |
| | T328 | 0,2960 |
| | T330 | 0,0600 |
| | T335 | 0,1576 |
| Figure 5F | | |
| TRF1-WT | T328 T330 T335 | 0,053 |
| | T328 | 0,2960 |
| | T330 | 0,0600 |
| | T335 | 0,1576 |

# Appendix Table S2: P Values

| Figure 4J | SAMPLE | P VALUE |
|---|---|---|
| | T4 | 0,04 |
| | T298 | 0,12 |
| | T330 | 0,02 |
| | T336 | 0,07 |
| Figure 4M | T4S6S7 | 0,00001 |
| | T44 | 0,02 |
| | T195 | 0,1 |
| | T268T270T274 | 0,0005 |
| | T298 | 0,87 |
| | T328T330T335 | 0,005 |
| | T358 | 0,17 |
| Figure 4N | T328 | 0,00001 |
| | T335 | 0,00001 |
| Figure 4O | T248 | 0,18 |
| | T330 | 0,04 |
| | S344 | 0,003 |
| | T330S344 | 0,0007 |
| **5G** | **WT+ERKi** | **0.04** |
| | T328+ERKi | 0.045 |
| | T330+ERKi | 0.03 |
| | T335+ERKi | 0.026 |
| | T328/T330/T335 +ERKi | 0.032 |
| 5H | Scrbl+ERKi | 0.03 |
| | WT+ERKi | 0.015 |
| | T328+ERKi | 0.024 |
| | T330+ERKi | 0.036 |
| | T335+ERKi | 0.041 |
| | T328/T330/T335 +ERKi | 0.039 |
| 6A | Scrbl | 0.008 |
| | shERK | 0.03 |
| | Scrbl | 0.004 |
| | shERK | 0.026 |
| 6B | ERK1/2 RNAi | 0.03 |
| | ERK1/2 RNAi | 0.021 |
| 6E | shERK | 0.03 |
| | shERK TRF1WT | 0.034 |
| | shERK T328 | 0.042 |
| | shERK T330 | 0.021 |
| | shERK T335 | 0.03 |
| | shERK T328/T330/T335 | 0.027 |

# Appendix Table S2: P Values

| Figure 7A | SAMPLE | P VALUE |
|---|---|---|
| DMSO | PI3Ki | 0,0001 |
| | RTKi | 7,34E-05 |
| | Combination | 2,09E-09 |
| Combination | PI3Ki | 5,50E-09 |
| | RTKi | 2,03E-05 |
| Figure 7B | | |
| DMSO | PI3Ki | 0,0022 |
| | ERKi | 0,1178 |
| | Combination | 3,89E-07 |
| Combination | PI3Ki | 2,03E-07 |
| | ERKi | 1,58E-07 |
| Figure 7C | | |
| DMSO | PI3Ki | 0,0011 |
| | MEKi | 0,0081 |
| | Combination | 1,26E-09 |
| Combination | PI3Ki | 3,80E-06 |
| | MEKi | 2,40E-06 |
| Figure 7D | | |
| DMSO | PI3Ki | 0,0190 |
| | HSP90i | 0,0002 |
| | Combination | 1,28E-06 |
| Combination | PI3Ki | 1,46E-07 |
| | HSP90i | 0,0028 |
| Figure 7E | | |
| DMSO | PI3Ki | 0,0018 |
| | Gemcitabine | 1,30E-05 |
| | Combination | 1,28E-07 |
| Combination | PI3Ki | 1,32E-09 |
| | Gemcitabine | 3,65E-05 |
| Figure 7F | | |
| DMSO | PI3Ki | 0,0064 |
| | Docetaxel | 1,63E-05 |
| | Combination | 1,00E-06 |
| Combination | PI3Ki | 7,14E-06 |
| | Docetaxel | 0,0034 |

# Appendix Table S2: P Values

| Figure 7G | SAMPLE | P VALUE |
|---|---|---|
| DMSO | PI3Ki | 0,0083 |
| | RTKi | 1,64E-05 |
| | Combination | 1,38E-06 |
| Combination | PI3Ki | 0,0052 |
| | RTKi | 0,0075 |
| | | |
| Figure 7H | | |
| DMSO | PI3Ki | 0,0083 |
| | ERKi | 0,0195 |
| | Combination | 0,0080 |
| Combination | PI3Ki | 0,2902 |
| | ERKi | 0,2932 |
| Figure 7I | | |
| DMSO | PI3Ki | 0,0083 |
| | MEKi | 0,0009 |
| | Combination | 8,35E-06 |
| Combination | PI3Ki | 0,0047 |
| | MEKi | 0,2955 |
| | | |
| Figure 7J | | |
| DMSO | PI3Ki | 0,0083 |
| | HSP90i | 0,0048 |
| | Combination | 2,03E-05 |
| Combination | PI3Ki | 0,0879 |
| | HSP90i | 0,6510 |
| | | |
| Figure 7K | | |
| DMSO | PI3Ki | 0,0083 |
| | Gemcitabine | 3,17E-05 |
| | Combination | 6,83E-07 |
| Combination | PI3Ki | 0,0751 |
| | Gemcitabine | 0,0048 |
| | | |
| Figure 7L | | |
| DMSO | PI3Ki | 0,0083 |
| | Docetaxel | 0,1271 |
| | Combination | 0,0035 |
| Combination | PI3Ki | 0,5163 |
| | Docetaxel | 0,7246 |

# Appendix Table S2: P Values

| | | |
|---|---|---|
| Figure 8A | | |
| Vehicle | PI3Ki | 1,22E-05 |
| | ERKi | 0,0210 |
| | Combination | 0,0120 |
| Combination | PI3Ki | 0,0507 |
| | | |
| Figure 8B | | |
| Vehicle | PI3Ki | 1,22E-05 |
| | MEKi | 0,0131 |
| | Combination | 0,0091 |
| Combination | PI3Ki | 0,0078 |
| | | |
| Figure 8C | | |
| Vehicle | PI3Ki | 1,22E-05 |
| | Docetaxel | 0,2138 |
| | Combination | 0,0037 |
| Combination | PI3Ki | 0,0390 |
| | | |
| Figure 8D | | |
| Vehicle | PI3Ki | 1,22E-05 |
| | Gemcitabine | 0,0292 |
| | Combination | 0,0013 |
| Combination | PI3Ki | 0,0570 |
| | | |
| Figure 8E | | |
| Vehicle | PI3Ki | 0,4186 |
| | ERKi | 0,1623 |
| | Combination | 0,0067 |
| PI3Ki | ERKi | 0,1812 |
| Combination | PI3Ki | 0,0780 |
| | ERKi | 0,0546 |
| | | |
| Figure 8F | | |
| Vehicle | PI3Ki | 0,4186 |
| | MEKi | 0,2206 |
| | Combination | 0,0069 |
| PI3Ki | MEKi | 0,1817 |
| Combination | PI3Ki | 0,0803 |
| | MEKi | 0,2365 |
| | | |
| Figure 8G | | |
| Vehicle | PI3Ki | 0,4186 |
| | Docetaxel | 0,0092 |
| | Combination | 0,0049 |
| PI3Ki | Docetaxel | 0,0868 |
| Combination | PI3Ki | 0,0732 |
| | Docetaxel | 0,1681 |
| | | |
| Figure 8H | | |
| Vehicle | PI3Ki | 0,4186 |
| | Gemcitabine | 0,7881 |
| | Combination | 0,9588 |
| PI3Ki | Gemcitabine | 0,5674 |
| Combination | PI3Ki | 0,4797 |
| | Gemcitabine | 0,8582 |

# Appendix Table S2: P Values

| Appendix figure S1A | SAMPLE | PVALUE |
|---|---|---|
| DMSO | Docetaxel | 0,3239 |
| | CDKi | 0,3785 |
| | ERKi | 0,0961 |
| | MEKi | 0,1092 |
| | RTKi | 0,0478 |
| | Gemcitabine | 0,4392 |
| | PI3Ki | 0,7605 |
| | HSP90i | 0,5911 |
| | mTORi | 0,5623 |
| | PLKi | 0,6030 |
| | Aurora | 0,8530 |
| | | |
| Appendix figure S1B | | |
| DMSO | Docetaxel | 0,0276 |
| | CDKi | 0,3845 |
| | ERKi | 0,5080 |
| | MEKi | 0,0280 |
| | RTKi | 0,0567 |
| | Gemcitabine | 0,2514 |
| | PI3Ki | 0,3827 |
| | HSP90i | 0,0566 |
| | mTORi | 0,5044 |
| | PLKi | 0,6370 |
| | Aurora | 0,3922 |
| | | |
| Appendix figure S2A | | |
| DMSO | PI3Ki | 0,2223 |
| | CDKi | 0,0002 |
| | Docetaxel | 0,0008 |
| | ERKi | 0,72 |
| | HSP90i | 0,1974 |
| | MEKi | 0,6095 |
| | mTORi | 0,5145 |
| | RTKi | 0,6513 |
| | | |
| Appendix figure S2B | | |
| DMSO | ERKi | 0,0003 |
| | MEKi | 0,0045 |
| | RTKi | 0,1113 |
| | HSP90i | 0,0044 |
| | CDKi | 0,1614 |
| | PI3Ki | 0,3842 |
| | Docetaxel | 0,0026 |
| | mTORi | 0,3270 |

# Appendix Table S2: P Values

| Appendix figure S2C | SAMPLE | P VALUE |
|---|---|---|
| DMSO | ERKi | 6,73E-05 |
| | PI3Ki | 0,0150 |
| | CDKi | 0,1128 |
| | Docetaxel | 0,6573 |
| | HSP90i | 0,0058 |
| | MEKi | 0,0094 |
| | mTORi | 0,0410 |
| | RTKi | 0,0224 |
| | | |
| Appendix figure S3A | | |
| DMSO | HSP90i | 0,0032 |
| | mTORi | 0,0073 |
| | Docetaxel | 0,7422 |
| | ERKi | 0,0595 |
| | MEKi | 0,2516 |
| | Gemcitabine | 0,0567 |
| | CDKi | 0,2645 |
| | RTKi | 0,0014 |
| | PI3Ki | 0,0215 |
| | | |
| Appendix figureS4 | P values are represented in the table below | |
| | | |
| Appendix figureS5A | | |
| Vehicle | PI3Ki | 0,48 |
| | PI3Ki+MEKi | 0,33 |
| | PI3Ki+Gemcitabine | 0,33 |
| | PI3Ki+Docetaxel | 0,01 |
| | PI3Ki+RTKi | 0,0047 |
| | PI3Ki+ERKi | 0,005 |
| | PI3Ki+HSP90i | 0,0016 |
| | | |
| Expanded View Figure 3C | | |
| For pAKT/AKT: Vehicle | PI3Ki (ETP-47037) | 0,8651 |
| For pS6/S6: Vehicle | PI3Ki (ETP-47037) | 0,6660 |
| | | |
| Expanded View Figure 3D | | |
| Vehicle | PI3Ki (ETP-47037) | 0,2514 |
| | | |
| Expanded View Figure 3E | | |
| Vehicle | PI3Ki (ETP-47037) | 0,4879 |
| | | |
| Expanded View Figure 4C | | |
| DMSO | PI3Ki | 1,56E-17 |
| | RTKi | 5,84E-45 |
| | Combination | 4,7E-73 |
| Combination | PI3Ki | 1,61E-58 |
| | RTKi | 9,74E-21 |

# Appendix Table S2: P Values

| | | |
|---|---|---|
| Expanded View Figure 4D | | |
| DMSO | PI3Ki | 1,00E-34 |
| | ERKi | 7,94E-12 |
| | Combination | 1,19E-62 |
| Combination | PI3Ki | 3,56E-35 |
| | ERKi | 6,86E-50 |
| | | |
| Expanded View Figure 4E | | |
| DMSO | PI3Ki | 0,0003 |
| | MEKi | 0,8340 |
| | Combination | 4,90E-23 |
| Combination | PI3Ki | 7,17E-17 |
| | MEKi | 5,23E-25 |
| Expanded View Figure 4F | | |
| DMSO | PI3Ki | 2,35E-20 |
| | HSP90i | 1,79E-32 |
| | Combination | 3,86E-35 |
| Combination | PI3Ki | 1,17E-18 |
| | HSP90i | 0,0005 |
| | | |
| Expanded View Figure 4G | | |
| DMSO | PI3Ki | 5,45E-07 |
| | Gemcitabine | 1,06E-21 |
| | Combination | 3,11E-22 |
| Combination | PI3Ki | 3,74E-17 |
| | Gemcitabine | 0,0009 |
| | | |
| Expanded View Figure 4H | | |
| DMSO | PI3Ki | 1,21E-10 |
| | Docetaxel | 1,97E-19 |
| | Combination | 1,94E-19 |
| Combination | PI3Ki | 1,39E-18 |
| | Docetaxel | 0,0002 |

# Appendix Table S2: P Values

| Appendix figure S4 | | | | | | |
|---|---|---|---|---|---|---|
| I) type | (J) type | Mean Difference (I-J) | Std. Error | Sig. | 95% Confidence Interval Lower Bound | 95% Confidence Interval Upper Bound |
| 0 | 1 | -4612.200* | 477.548 | .000 | -6330.19 | -2894.21 |
| | 44 | -5108.000* | 477.548 | .000 | -6825.99 | -3390.01 |
| | 195 | -4376.800* | 477.548 | .000 | -6094.79 | -2658.81 |
| | 298 | -4885.400* | 477.548 | .000 | -6603.39 | -3167.41 |
| | 328 | -4517.200* | 477.548 | .000 | -6235.19 | -2799.21 |
| | 330 | 766.600 | 477.548 | 1.000 | -951.39 | 2484.59 |
| | 335 | -2829.400* | 477.548 | .000 | -4547.39 | -1111.41 |
| | 467 | -3408.400* | 477.548 | .000 | -5126.39 | -1690.41 |
| | 358 | -3292.400* | 477.548 | .000 | -5010.39 | -1574.41 |
| | 3268 | -4375.400* | 477.548 | .000 | -6093.39 | -2657.41 |
| | 3328 | -3533.800* | 477.548 | .000 | -5251.79 | -1815.81 |
| 1 | 0 | 4612.200* | 477.548 | .000 | 2894.21 | 6330.19 |
| | 44 | -495.800 | 477.548 | 1.000 | -2213.79 | 1222.19 |
| | 195 | 235.400 | 477.548 | 1.000 | -1482.59 | 1953.39 |
| | 298 | -273.200 | 477.548 | 1.000 | -1991.19 | 1444.79 |
| | 328 | 95.000 | 477.548 | 1.000 | -1622.99 | 1812.99 |
| | 330 | 5378.800* | 477.548 | .000 | 3660.81 | 7096.79 |
| | 335 | 1782.800* | 477.548 | .033 | 64.81 | 3500.79 |
| | 467 | 1203.800 | 477.548 | .996 | -514.19 | 2921.79 |
| | 358 | 1319.800 | 477.548 | .533 | -398.19 | 3037.79 |
| | 3268 | 236.800 | 477.548 | 1.000 | -1481.19 | 1954.79 |
| | 3328 | 1078.400 | 477.548 | 1.000 | -639.59 | 2796.39 |
| 44 | 0 | 5108.000* | 477.548 | .000 | 3390.01 | 6825.99 |
| | 1 | 495.800 | 477.548 | 1.000 | -1222.19 | 2213.79 |
| | 195 | 731.200 | 477.548 | 1.000 | -986.79 | 2449.19 |
| | 298 | 222.600 | 477.548 | 1.000 | -1495.39 | 1940.59 |
| | 328 | 590.800 | 477.548 | 1.000 | -1127.19 | 2308.79 |
| | 330 | 5874.600* | 477.548 | .000 | 4156.61 | 7592.59 |
| | 335 | 2278.600* | 477.548 | .001 | 560.61 | 3996.59 |
| | 467 | 1699.600 | 477.548 | .056 | -18.39 | 3417.59 |
| | 358 | 1815.600* | 477.548 | .027 | 97.61 | 3533.59 |
| | 3268 | 732.600 | 477.548 | 1.000 | -985.39 | 2450.59 |
| | 3328 | 1574.200 | 477.548 | .122 | -143.79 | 3292.19 |
| 195 | 0 | 4376.800* | 477.548 | .000 | 2658.81 | 6094.79 |
| | 1 | -235.400 | 477.548 | 1.000 | -1953.39 | 1482.59 |
| | 44 | -731.200 | 477.548 | 1.000 | -2449.19 | 986.79 |
| | 298 | -508.600 | 477.548 | 1.000 | -2226.59 | 1209.39 |
| | 328 | -140.400 | 477.548 | 1.000 | -1858.39 | 1577.59 |
| | 330 | 5143.400* | 477.548 | .000 | 3425.41 | 6861.39 |
| | 335 | 1547.400 | 477.548 | .143 | -170.59 | 3265.39 |
| | 467 | 968.400 | 477.548 | 1.000 | -749.59 | 2686.39 |
| | 358 | 1084.400 | 477.548 | 1.000 | -633.59 | 2802.39 |
| | 3268 | 1.400 | 477.548 | 1.000 | -1716.59 | 1719.39 |
| | 3328 | 843.000 | 477.548 | 1.000 | -874.99 | 2560.99 |
| 298 | 0 | 4885.400* | 477.548 | .000 | 3167.41 | 6603.39 |
| | 1 | 273.200 | 477.548 | 1.000 | -1444.79 | 1991.19 |
| | 44 | -222.600 | 477.548 | 1.000 | -1940.59 | 1495.39 |
| | 195 | 508.600 | 477.548 | 1.000 | -1209.39 | 2226.59 |
| | 328 | 368.200 | 477.548 | 1.000 | -1349.79 | 2086.19 |
| | 330 | 5652.000* | 477.548 | .000 | 3934.01 | 7369.99 |
| | 335 | 2056.000* | 477.548 | .005 | 338.01 | 3773.99 |
| | 467 | 1477.000 | 477.548 | .218 | -240.99 | 3194.99 |
| | 358 | 1593.000 | 477.548 | .109 | -124.99 | 3310.99 |
| | 3268 | 510.000 | 477.548 | 1.000 | -1207.99 | 2227.99 |
| | 3328 | 1351.600 | 477.548 | .447 | -366.39 | 3069.59 |

# Appendix Table S2: P Values

| | | | | | | | | |
|---|---|---|---|---|---|---|---|---|
| | 328 | dimension3 | 0 | 4517.200* | 477.548 | .000 | 2799.21 | 6235.19 |
| | | | 1 | -95.000 | 477.548 | 1.000 | -1812.99 | 1622.99 |
| | | | 44 | -590.800 | 477.548 | 1.000 | -2308.79 | 1127.19 |
| | | | 195 | 140.400 | 477.548 | 1.000 | -1577.59 | 1858.39 |
| | | | 298 | -368.200 | 477.548 | 1.000 | -2086.19 | 1349.79 |
| | | | 330 | 5283.800* | 477.548 | .000 | 3565.81 | 7001.79 |
| | | | 335 | 1687.800 | 477.548 | .060 | -30.19 | 3405.79 |
| | | | 467 | 1108.800 | 477.548 | 1.000 | -609.19 | 2826.79 |
| | | | 358 | 1224.800 | 477.548 | .891 | -493.19 | 2942.79 |
| | | | 3268 | 141.800 | 477.548 | 1.000 | -1576.19 | 1859.79 |
| | | | 3328 | 983.400 | 477.548 | 1.000 | -734.59 | 2701.39 |
| | 330 | dimension3 | 0 | -766.600 | 477.548 | 1.000 | -2484.59 | 951.39 |
| | | | 1 | -5378.800* | 477.548 | .000 | -7096.79 | -3660.81 |
| | | | 44 | -5874.600* | 477.548 | .000 | -7592.59 | -4156.61 |
| | | | 195 | -5143.400* | 477.548 | .000 | -6861.39 | -3425.41 |
| | | | 298 | -5652.000* | 477.548 | .000 | -7369.99 | -3934.01 |
| | | | 328 | -5283.800* | 477.548 | .000 | -7001.79 | -3565.81 |
| | | | 335 | -3596.000* | 477.548 | .000 | -5313.99 | -1878.01 |
| | | | 467 | -4175.000* | 477.548 | .000 | -5892.99 | -2457.01 |
| | | | 358 | -4059.000* | 477.548 | .000 | -5776.99 | -2341.01 |
| | | | 3268 | -5142.000* | 477.548 | .000 | -6859.99 | -3424.01 |
| | | | 3328 | -4300.400* | 477.548 | .000 | -6018.39 | -2582.41 |
| | 335 | dimension3 | 0 | 2829.400* | 477.548 | .000 | 1111.41 | 4547.39 |
| | | | 1 | -1782.800* | 477.548 | .033 | -3500.79 | -64.81 |
| | | | 44 | -2278.600* | 477.548 | .001 | -3996.59 | -560.61 |
| | | | 195 | -1547.400 | 477.548 | .143 | -3265.39 | 170.59 |
| | | | 298 | -2056.000* | 477.548 | .005 | -3773.99 | -338.01 |
| | | | 328 | -1687.800 | 477.548 | .060 | -3405.79 | 30.19 |
| | | | 330 | 3596.000* | 477.548 | .000 | 1878.01 | 5313.99 |
| | | | 467 | -579.000 | 477.548 | 1.000 | -2296.99 | 1138.99 |
| | | | 358 | -463.000 | 477.548 | 1.000 | -2180.99 | 1254.99 |
| | | | 3268 | -1546.000 | 477.548 | .145 | -3263.99 | 171.99 |
| | | | 3328 | -704.400 | 477.548 | 1.000 | -2422.39 | 1013.59 |
| | 4_6_7 | dimension3 | 0 | 3408.400* | 477.548 | .000 | 1690.41 | 5126.39 |
| | | | 1 | -1203.800 | 477.548 | .996 | -2921.79 | 514.19 |
| | | | 44 | -1699.600 | 477.548 | .056 | -3417.59 | 18.39 |
| | | | 195 | -968.400 | 477.548 | 1.000 | -2686.39 | 749.59 |
| | | | 298 | -1477.000 | 477.548 | .218 | -3194.99 | 240.99 |
| | | | 328 | -1108.800 | 477.548 | 1.000 | -2826.79 | 609.19 |
| | | | 330 | 4175.000* | 477.548 | .000 | 2457.01 | 5892.99 |
| | | | 335 | 579.000 | 477.548 | 1.000 | -1138.99 | 2296.99 |
| | | | 358 | 116.000 | 477.548 | 1.000 | -1601.99 | 1833.99 |
| | | | 3268 | -967.000 | 477.548 | 1.000 | -2684.99 | 750.99 |
| | | | 3328 | -125.400 | 477.548 | 1.000 | -1843.39 | 1592.59 |
| | 358 | dimension3 | 0 | 3292.400* | 477.548 | .000 | 1574.41 | 5010.39 |
| | | | 1 | -1319.800 | 477.548 | .533 | -3037.79 | 398.19 |
| | | | 44 | -1815.600* | 477.548 | .027 | -3533.59 | -97.61 |
| | | | 195 | -1084.400 | 477.548 | 1.000 | -2802.39 | 633.59 |
| | | | 298 | -1593.000 | 477.548 | .109 | -3310.99 | 124.99 |
| | | | 328 | -1224.800 | 477.548 | .891 | -2942.79 | 493.19 |
| | | | 330 | 4059.000* | 477.548 | .000 | 2341.01 | 5776.99 |
| | | | 335 | 463.000 | 477.548 | 1.000 | -1254.99 | 2180.99 |
| | | | 467 | -116.000 | 477.548 | 1.000 | -1833.99 | 1601.99 |
| | | | 3268 | -1083.000 | 477.548 | 1.000 | -2800.99 | 634.99 |
| | | | 3328 | -241.400 | 477.548 | 1.000 | -1959.39 | 1476.59 |
| | 3268 | dimension3 | 0 | 4375.400* | 477.548 | .000 | 2657.41 | 6093.39 |
| | | | 1 | -236.800 | 477.548 | 1.000 | -1954.79 | 1481.19 |
| | | | 44 | -732.600 | 477.548 | 1.000 | -2450.59 | 985.39 |
| | | | 195 | -1.400 | 477.548 | 1.000 | -1719.39 | 1716.59 |
| | | | 298 | -510.000 | 477.548 | 1.000 | -2227.99 | 1207.99 |
| | | | 328 | -141.800 | 477.548 | 1.000 | -1859.79 | 1576.19 |
| | | | 330 | 5142.000* | 477.548 | .000 | 3424.01 | 6859.99 |
| | | | 335 | 1546.000 | 477.548 | .145 | -171.99 | 3263.99 |
| | | | 356 | 967.000 | 477.548 | 1.000 | -750.99 | 2684.99 |
| | | | 368 | 1083.000 | 477.548 | 1.000 | -634.99 | 2800.99 |
