# Supplementary material for: Multiple cancer pathways regulate telomere protection
Source: EMBO Mol Med. 2019 Jun 13;11(7):e10292. doi: 10.15252/emmm.201910292 (PMC6609915; doi:10.15252/emmm.201910292)
Supplement: Supplementary file 2 — Expanded View Figures PDF [file EMMM-11-e10292-s002.pdf]

Expanded View Figures

Figure EV1. Validation of the novel pathways of TRF1 regulation.

A Representative image of the pathways included in Reactome database.
B Quantification of TRF1 nuclear fluorescence in CHA9-3 lung cancer cells treated with DMSO or structurally different MEK inhibitors for 24 h at 1 μM. Data are representative of $n = 2$ biological replicates
C Quantification of TRF1 nuclear fluorescence in CHA9-3 lung cancer cells treated with DMSO or structurally different ERK inhibitors for 24 h at 1 μM. Data are representative of $n = 2$ biological replicates
D Western blot images (up) and p-ERK protein levels (down) of CHA9-3 lung cancer cells treated with the indicated compounds for 24 h at 1 μM. Data are representative of $n = 2$ biological replicates
E Quantification of TRF1 nuclear fluorescence in CHA9-3 lung cancer cells treated with DMSO or structurally different HSP90 inhibitors for 24 h at 1 μM. Data are representative of $n = 2$ biological replicates
F Quantification of TRF1 nuclear fluorescence in CHA9-3 lung cancer cells treated with DMSO or different tubulin agents for 24 h at 1 μM. Data are representative of $n = 2$ biological replicates.

Data information: Data are represented as mean ± SEM. Significant differences using unpaired t-test are indicated by *$P < 0.05$, **$P < 0.01$, ***$P < 0.001$.

Source data are available online for this figure.

▶

© 2019 The Authors

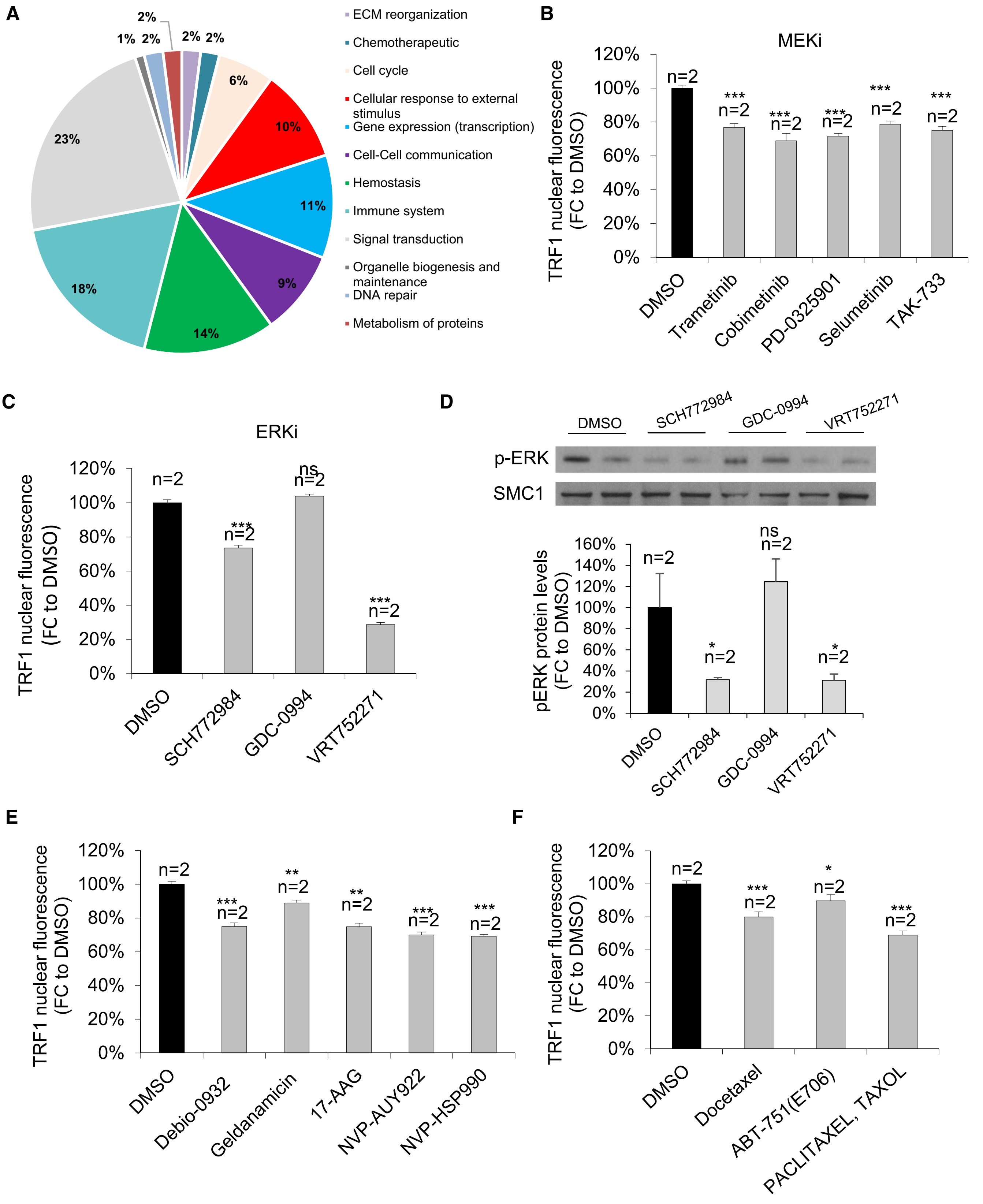
A
2%
1% 2%
2% 2%
6%
10%
11%
9%
14%
18%
23%
ECM reorganization
Chemotherapeutic
Cell cycle
Cellular response to external stimulus
Gene expression (transcription)
Cell-Cell communication
Hemostasis
Immune system
Signal transduction
Organelle biogenesis and maintenance
DNA repair
Metabolism of proteins
B
MEKi
TRF1 nuclear fluorescence (FC to DMSO)
120%
100%
80%
60%
40%
20%
0%
n=2
***
DMSO
Trametinib
Cobimetinib
PD-0325901
Selumetinib
TAK-733
C
ERKi
ns
SCH772984
GDC-0994
VRT752271
D
p-ERK
SMC1
pERK protein levels (FC to DMSO)
160%
140%
*
E
**
Debio-0932
Geldanamicin
17-AAG
NVP-AUY922
NVP-HSP990
F
Docetaxel
ABT-751(E706)
PACLITAXEL, TAXOL

Figure EV1.

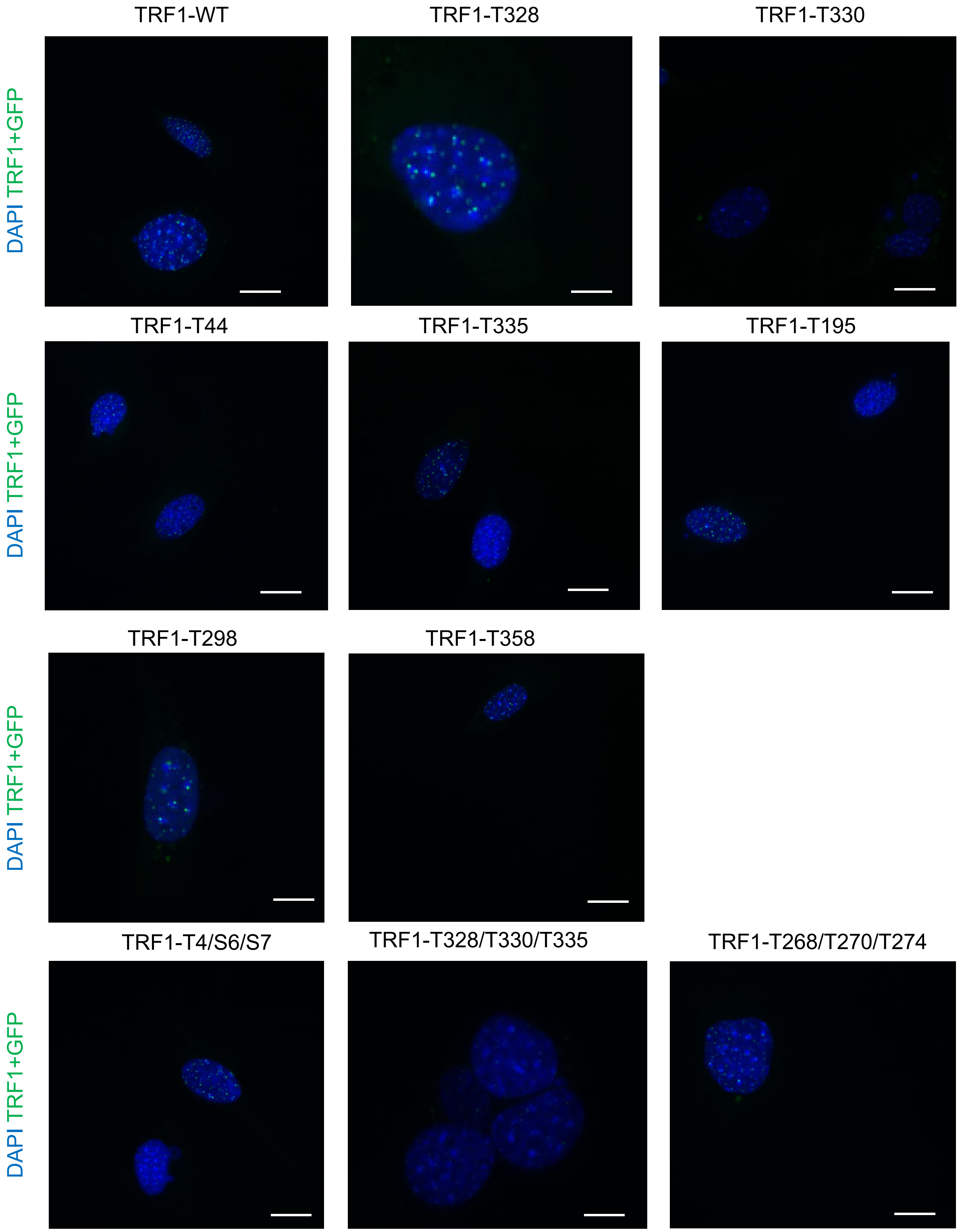

Figure EV2. GFP-tagged TRF1 telomeric foci in MEFs expressing either WT or mutants in ERK-dependent phosphosites of GFP-TRF1.

Representative images of *Trf1*$^{\Delta/\Delta}$ MEFs transduced with eGFP-*Trf1* WT or mutant alleles as indicated. Scale bars, 5 µm.

 © 2019 The Authors

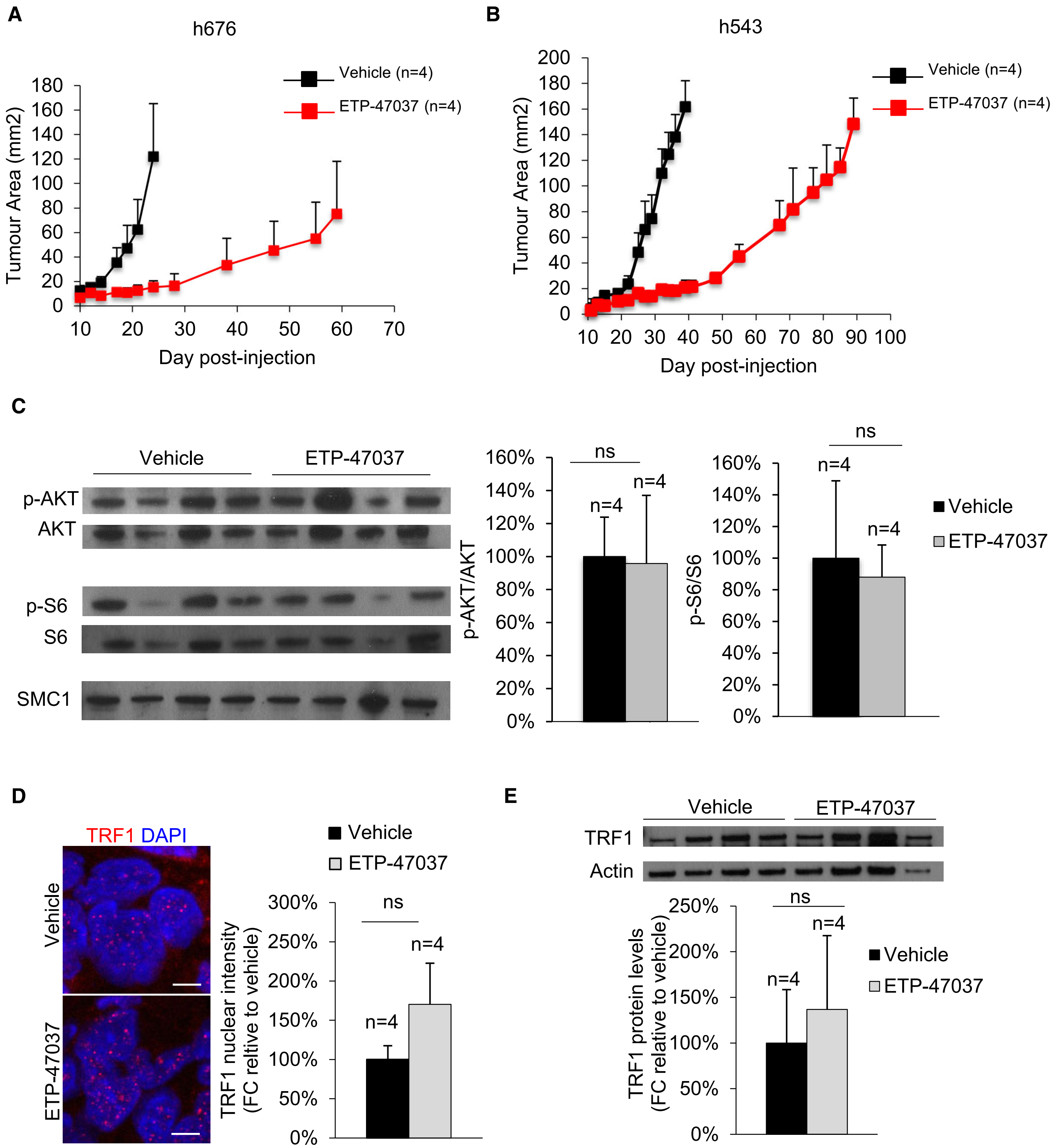
A
h676
Vehicle (n=4)
ETP-47037 (n=4)
Tumour Area (mm2)
Day post-injection
B
h543
Vehicle (n=4)
ETP-47037 (n=4)
Tumour Area (mm2)
Day post-injection
C
Vehicle
ETP-47037
p-AKT
AKT
p-S6
S6
SMC1
p-AKT/AKT
p-S6/S6
ns
n=4
Vehicle
ETP-47037
D
TRF1 DAPI
Vehicle
ETP-47037
TRF1 nuclear intensity (FC reltive to vehicle)
ns
n=4
E
Vehicle
ETP-47037
TRF1
Actin
TRF1 protein levels (FC relative to vehicle)
ns
n=4

Figure EV3.

Figure EV3. Patient-derived xenografts become resistant to PI3Ki.

A Longitudinal tumor growth follow-up in ETP-47037 or vehicle-treated mice injected with patient-derived h676 GSCs. Data are representative of n = 4 independent tumors.

B Longitudinal tumor growth follow-up in ETP-47037 or vehicle-treated mice injected with patient derived h543 GSCs. Data are representative of n = 4 independent tumors.

C Western blot images (left) and p-AKT/AKT or pS6/S6 protein levels (right) in vehicle- or ETP-47037-treated tumors. Data are representative of n = 4 independent tumors.

D TRF1 nuclear fluorescence in ETP-47037 or vehicle-treated tumors. Scale bars, 10 µm. Data are representative of n = 4 independent tumors

E Western blot images (left) and TRF1 protein levels (right) in vehicle- or ETP-47037-treated tumors. Data are representative of n = 4 independent tumors.

Data information: Data are represented as mean ± SEM.

Source data are available online for this figure.

Figure EV4. *In vitro* combinatorial studies with the new TRF1 inhibitory compounds.

A, B Number of spheres formed by patient-derived h676 GSCs 7 days after treatment with the indicated compounds as single agents or in combination. Data are representative of n = 2 biological replicates

C–H Diameter of spheres formed by patient-derived h676 GSCs 7 days after treatment with the indicated compounds as single agents or in combination. n represents biological replicates: in (C) DMSO n = 42, PI3Ki n = 49, RTKi n = 56, Comb n = 82; in (D) DMSO n = 48, PI3Ki n = 57; ERKi n = 48, Comb n = 71; in (E) DMSO n = 50; PI3Ki n = 49, MEKi n = 45, Comb n = 47; in (F) DMSO n = 39, PI3Ki n = 38, HSP90i n = 34, Comb n = 31; in (G) DMSO n = 42, PI3Ki n = 35, Gem n = 32, Comb n = 28; in (H) DMSO n = 37, PI3Ki n = 35, Doc n = 34, Comb n = 30.

Data information: Data are represented as mean ± SEM. Significant differences using unpaired t-test are indicated by ***$P < 0.001$.

© 2019 The Authors

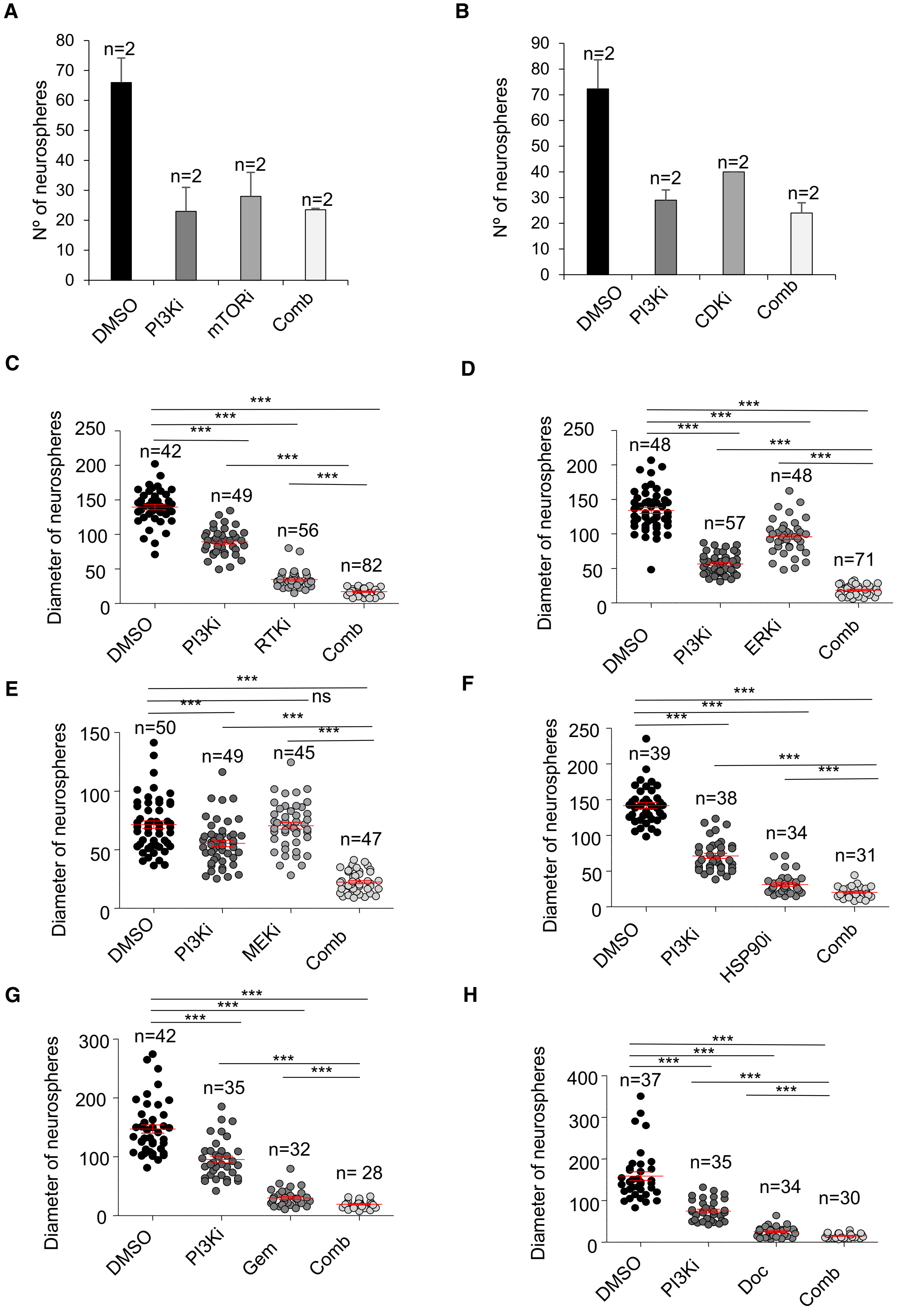

Figure EV4.

© 2019 The Authors
