# Supplementary material for: Multiple cancer pathways regulate telomere protection
Source: EMBO Mol Med. 2019 Jun 13;11(7):e10292. doi: 10.15252/emmm.201910292 (PMC6609915; doi:10.15252/emmm.201910292)
Supplement: Supplementary file 3 — Source Data for Expanded View and Appendix [file EMMM-11-e10292-s008.zip › EV_Appendix_SD/EMM-2019-10292-V2_Source_Data_Appendix_FigS1_1558003436_2.pdf]

Source Data Appendix Figure S1

DMSO
Doc
CDKi
ERKi
MEKi
RTKi

DMSO
Gem
PI3K
HSP90i
mTORi
PLKi
Aurorai

A

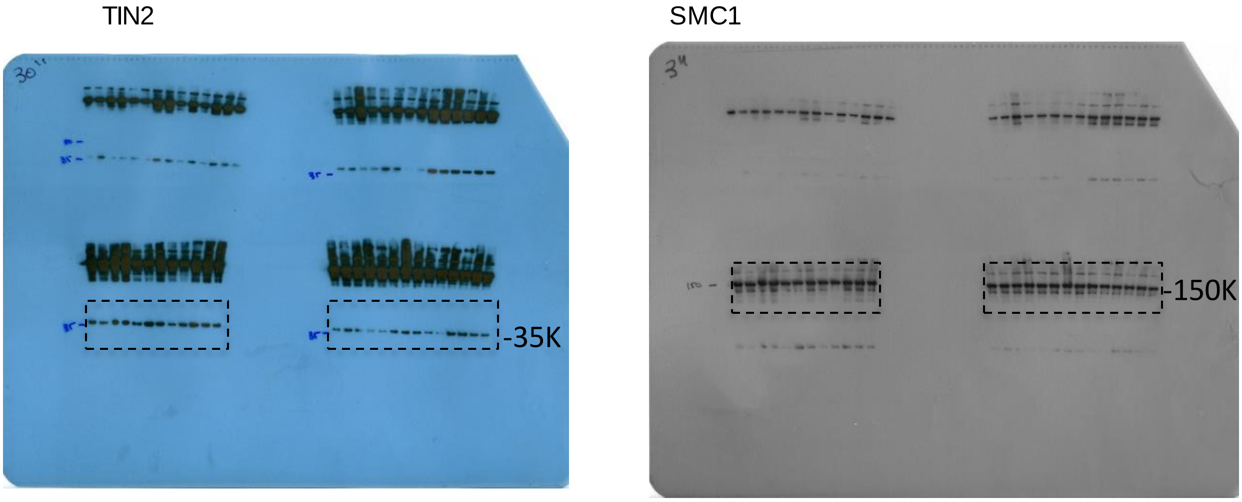

B

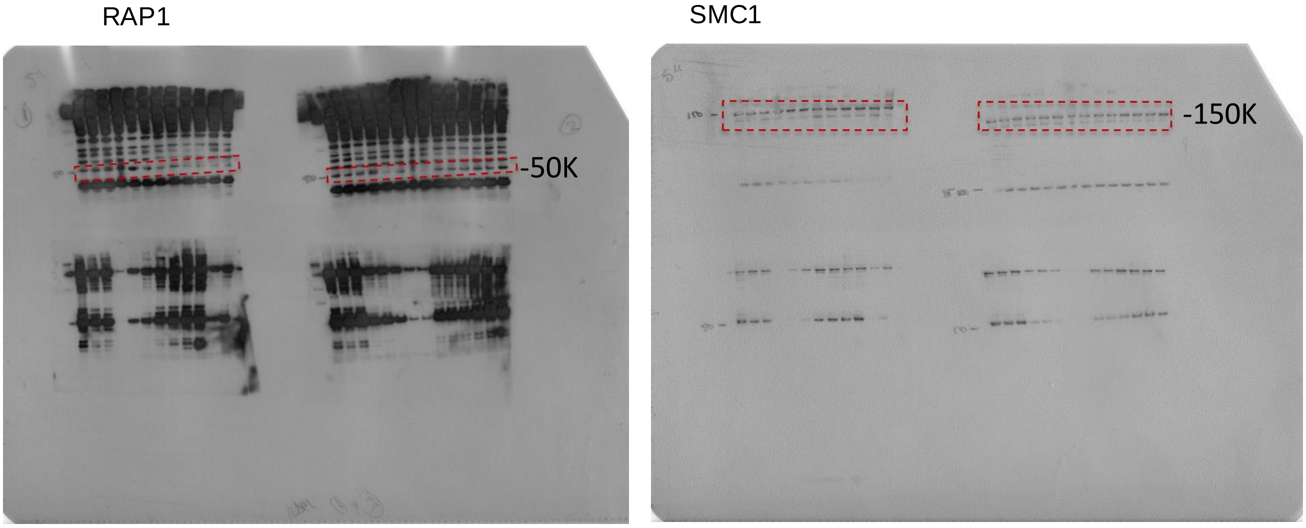

DMSO
Doc
CDKi
ERKi
MEKi
RTKi

DMSO
Gem
PI3K
HSP90i
mTORi
PLKi
Aurorai
