# Supplementary figures and images for: Multiple cancer pathways regulate telomere protection
Source: EMBO Mol Med. 2019 Jun 13;11(7):e10292. doi: 10.15252/emmm.201910292 (PMC6609915; doi:10.15252/emmm.201910292)

Vehicle x4    47037 x4

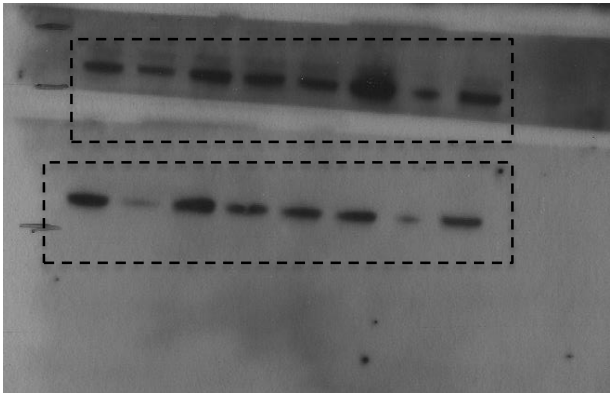

60 kDa    pAKT  
32 kDa    pS6

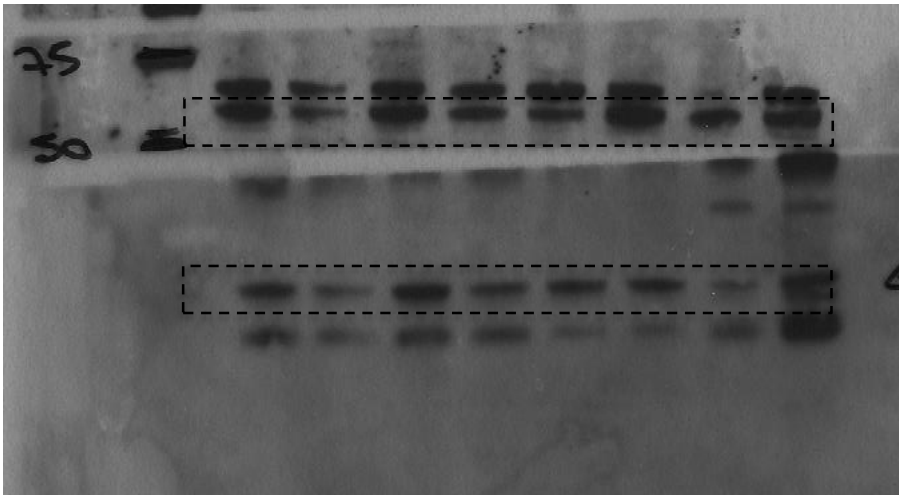

60 kDa    AKT  
32 kDa    S6

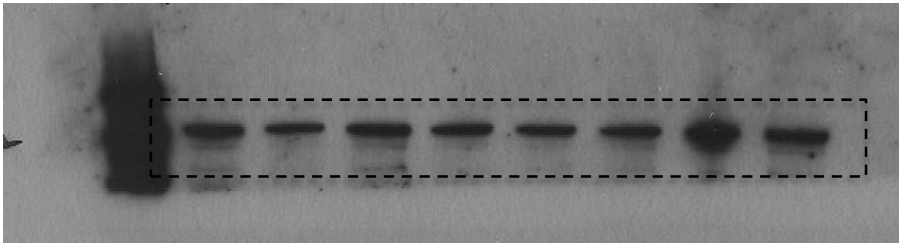

150 kDa    SMC1

Supplement: Supplementary file 3 — Source Data for Expanded View and Appendix [file EMMM-11-e10292-s008.zip › EV_Appendix_SD/EMM-2019-10292-V2_Source_Data_EV_Fig3.pdf]

pAKT (60 kDa)

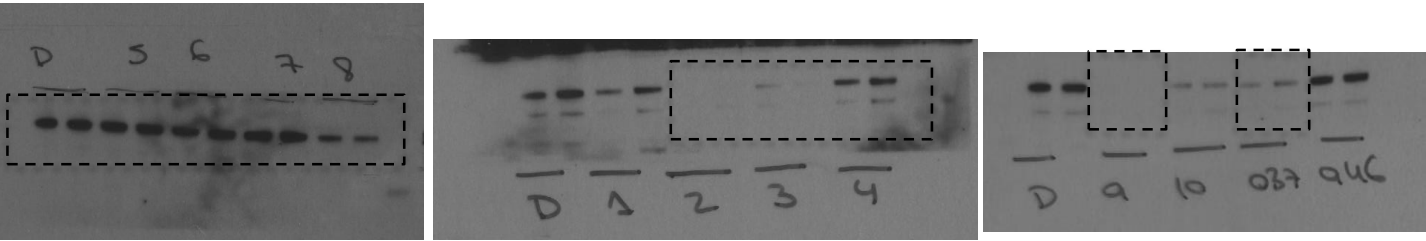

AKT total (60 kDa)

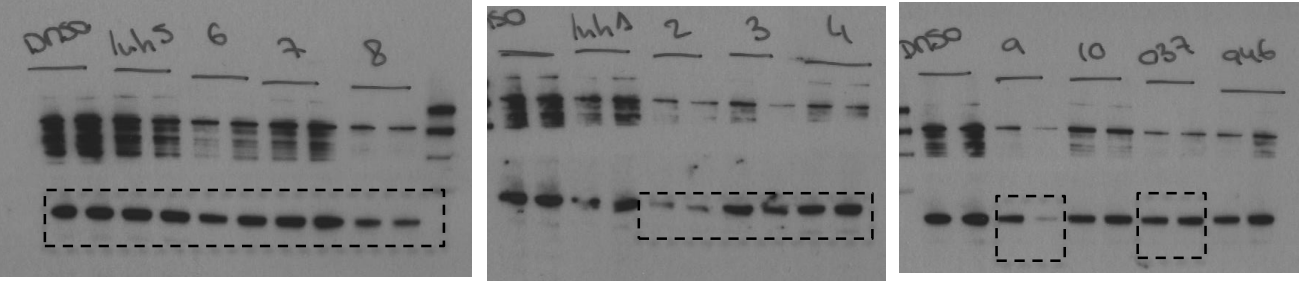

Actin (37 kDa)

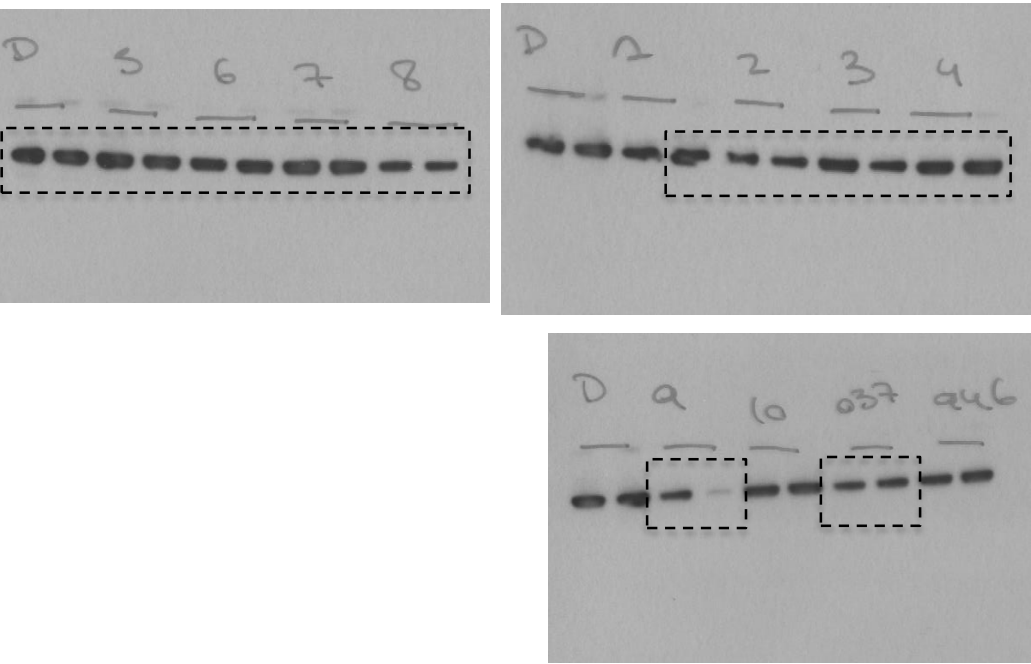

- 1 – Aurorai
- 2-HSP90
- 3-mTOR
- 4-Doc
- 5-Erk
- 6-Mek
- 7-Gem
- 8-CDK
- 9-RTK
- 10-PLK
- 037-PI3K

Supplement: Supplementary file 3 — Source Data for Expanded View and Appendix [file EMMM-11-e10292-s008.zip › EV_Appendix_SD/EMM-2019-10292-V2_Source_Data_Appendix_FigS3.pdf]

1B

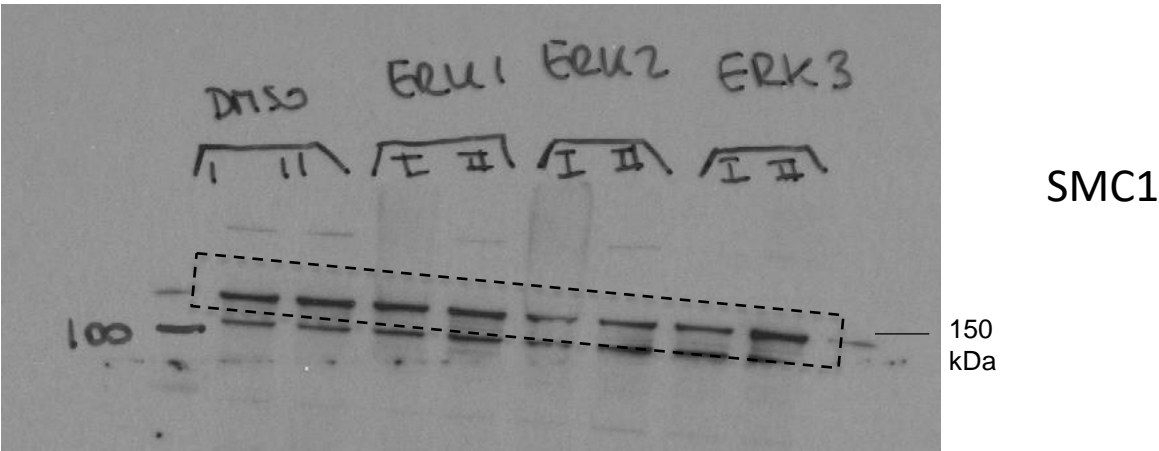

P-ERK

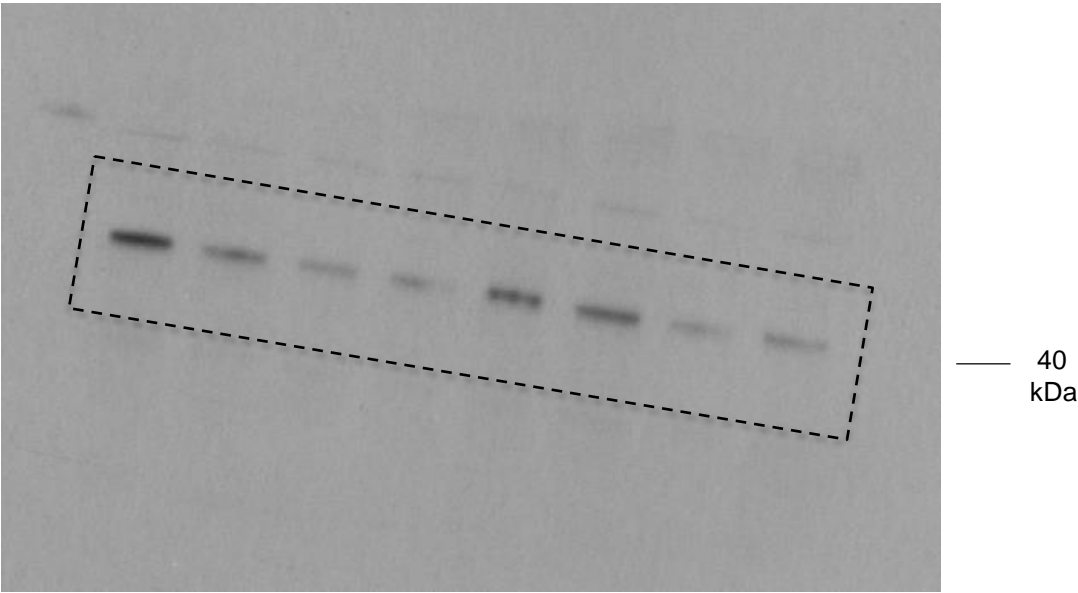

Supplement: Supplementary file 3 — Source Data for Expanded View and Appendix [file EMMM-11-e10292-s008.zip › EV_Appendix_SD/EMM-2019-10292-V2_Source_Data_EV_Fig1B.pdf]

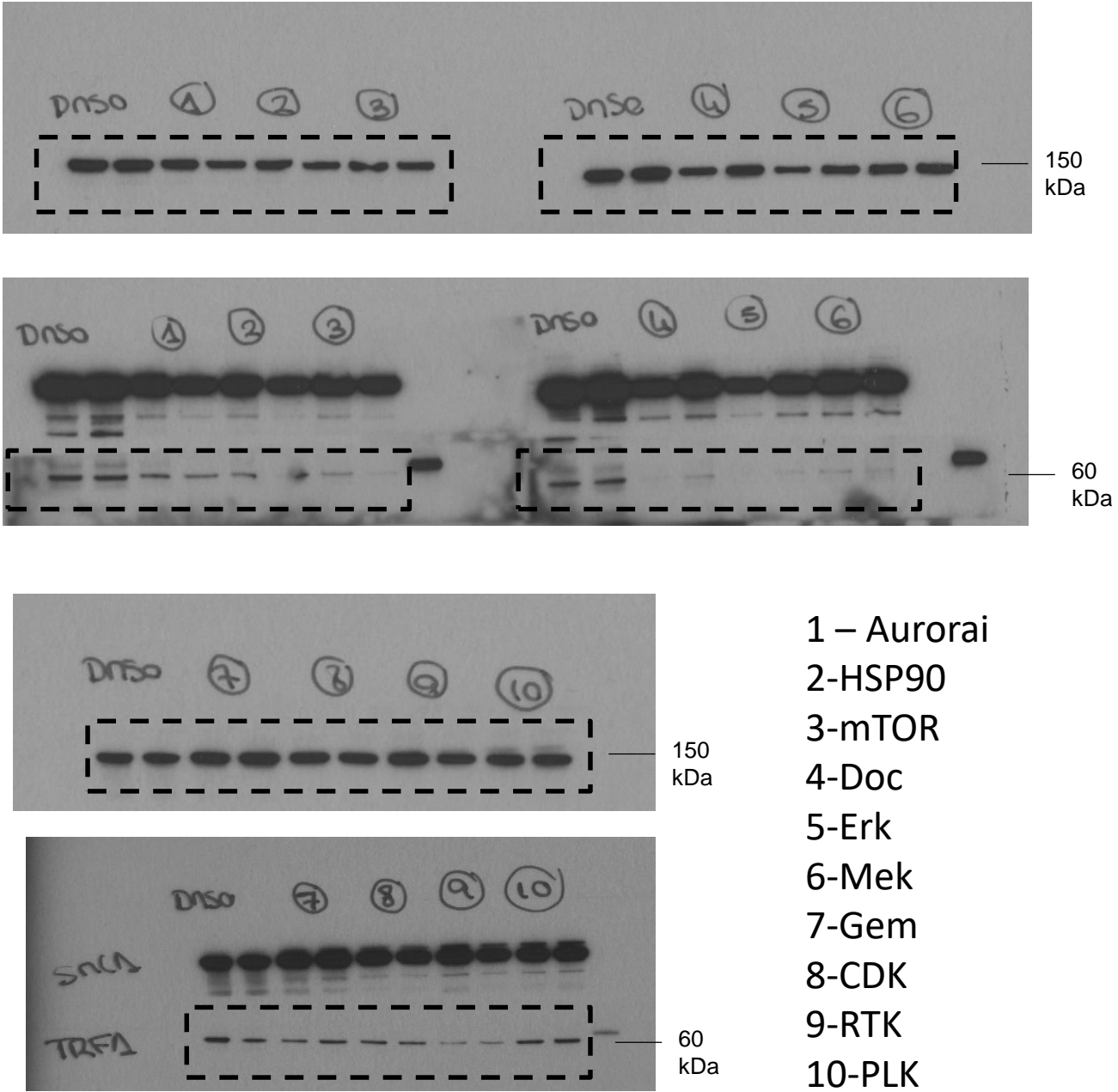

Supplement: Supplementary file 5 — Source Data for Figure 1 [file EMMM-11-e10292-s003.pdf]

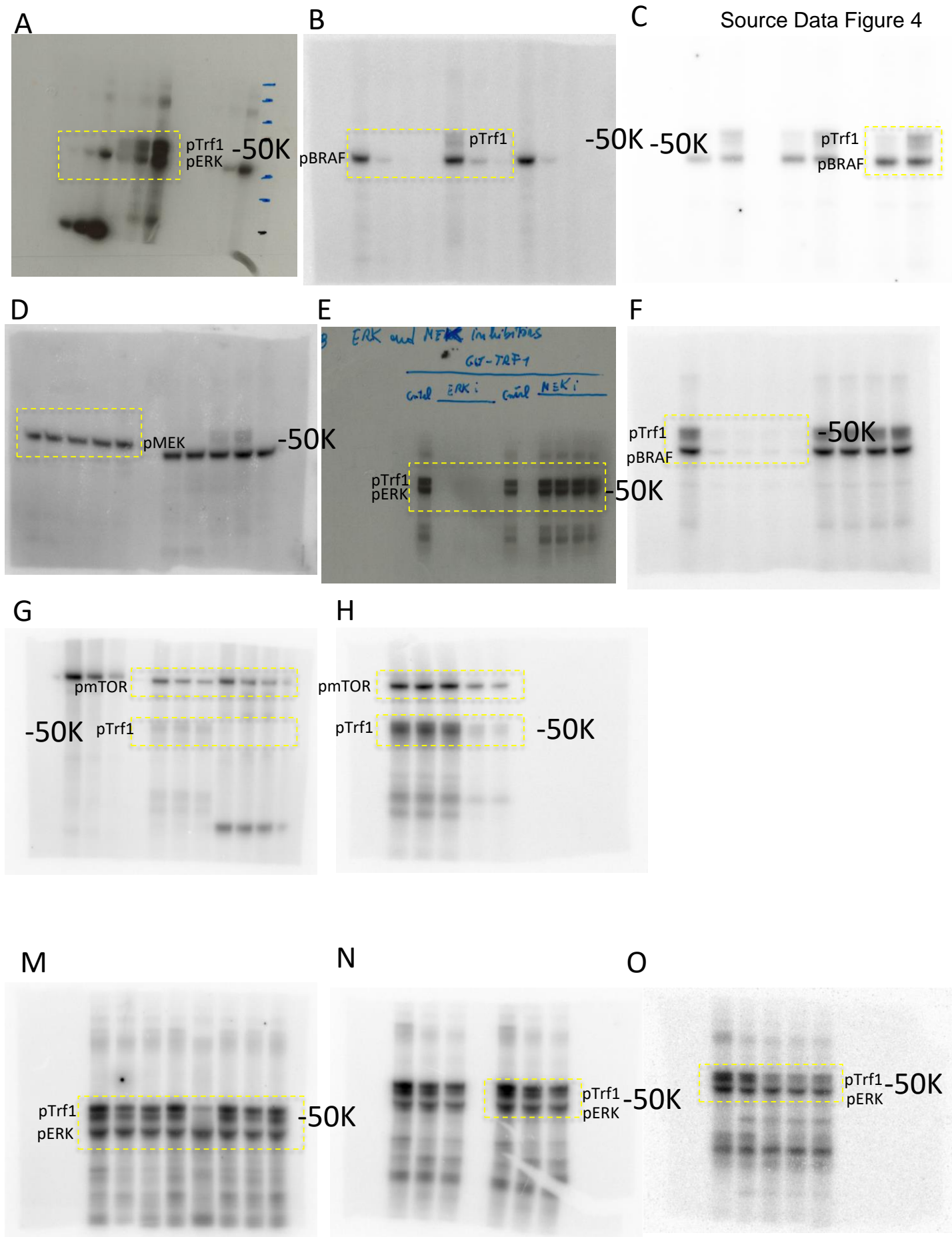

Supplement: Supplementary file 6 — Source Data for Figure 4 [file EMMM-11-e10292-s004.pdf]

B

SMC1

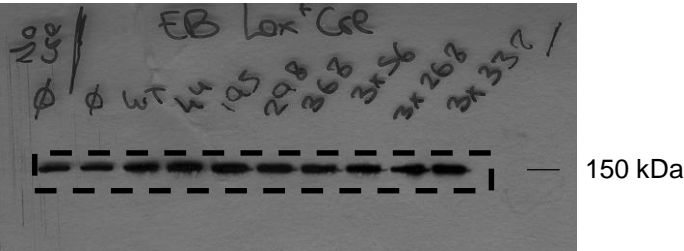

TRF1 GFP

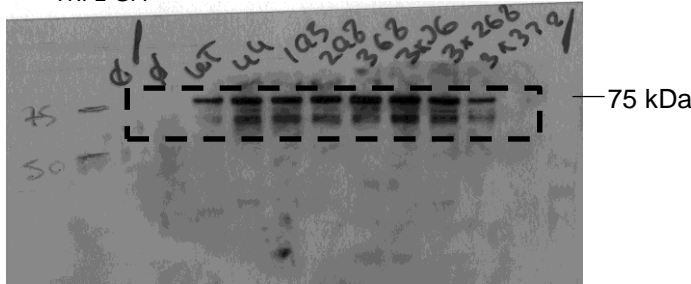

TRF1

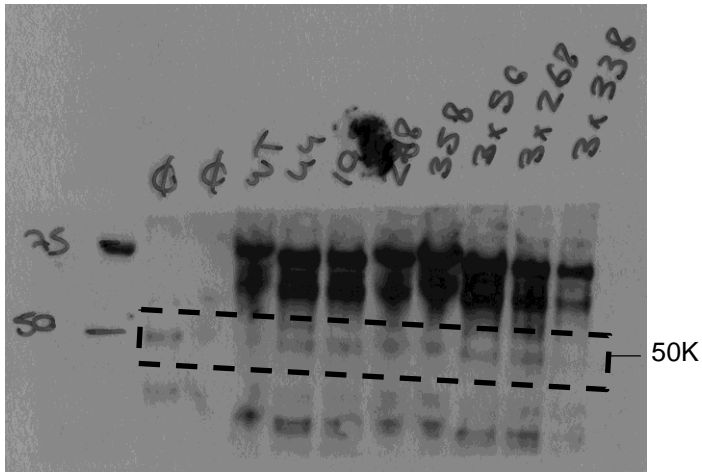

E

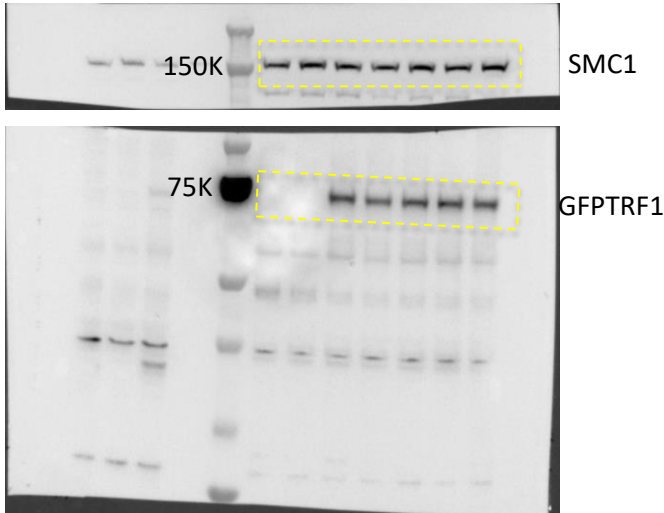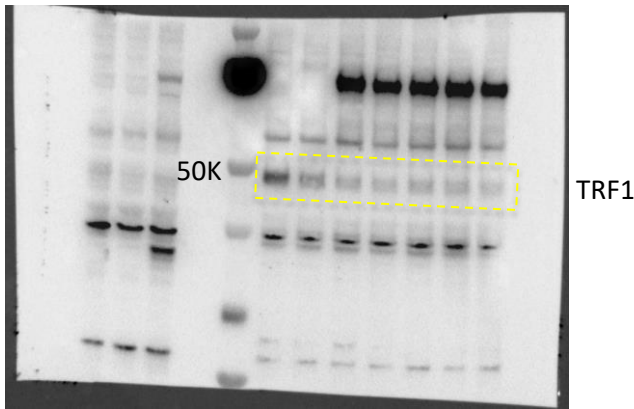

H

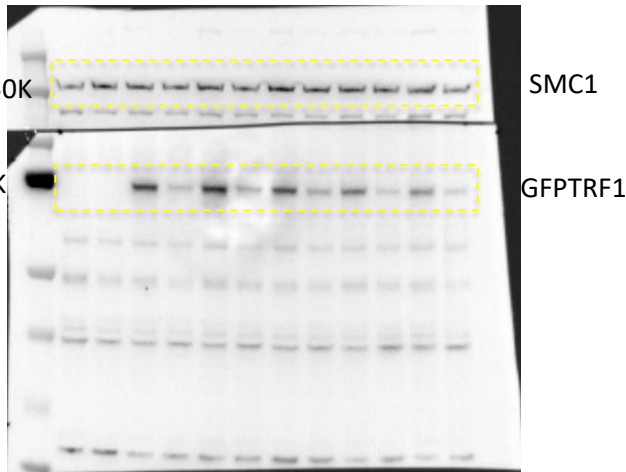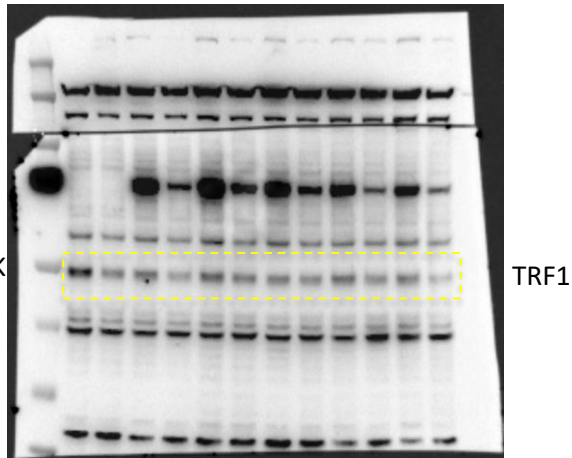

Supplement: Supplementary file 7 — Source Data for Figure 5 [file EMMM-11-e10292-s005.pdf]

Source Data Figure 6

A

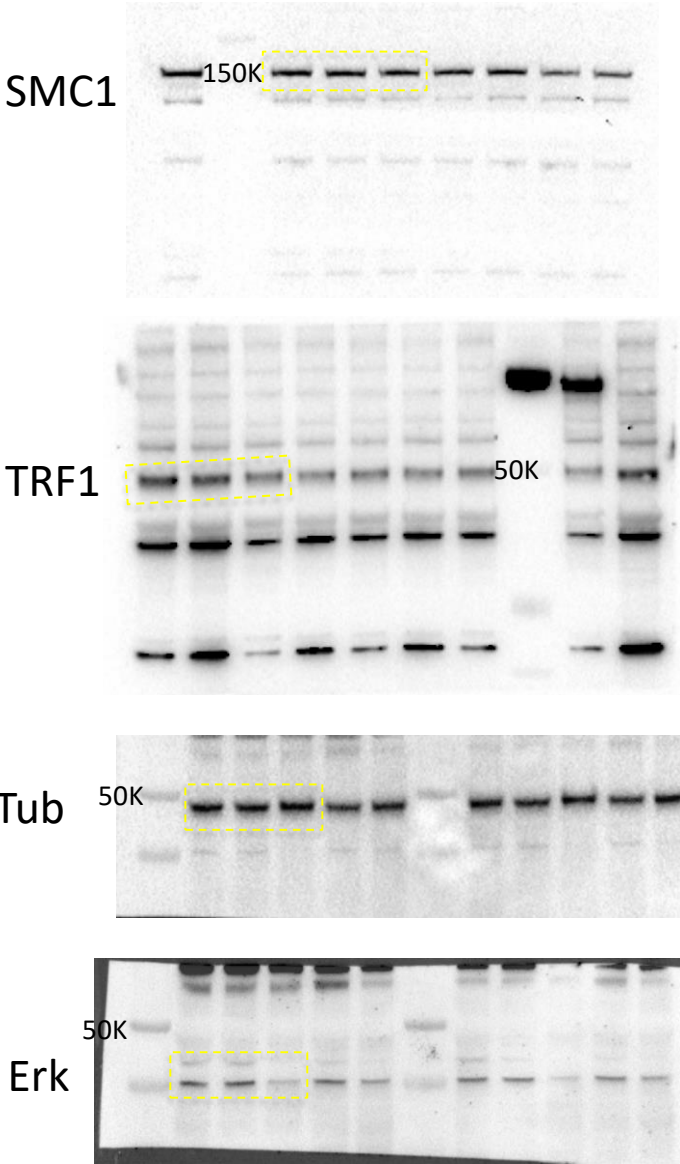

D

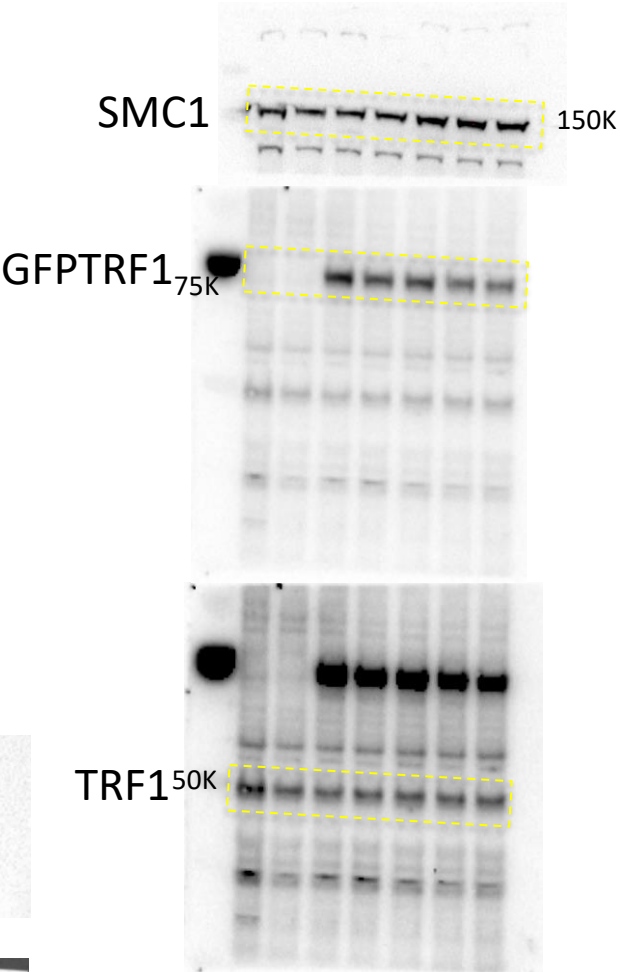

Supplement: Supplementary file 8 — Source Data for Figure 6 [file EMMM-11-e10292-s006.pdf]

SMC1

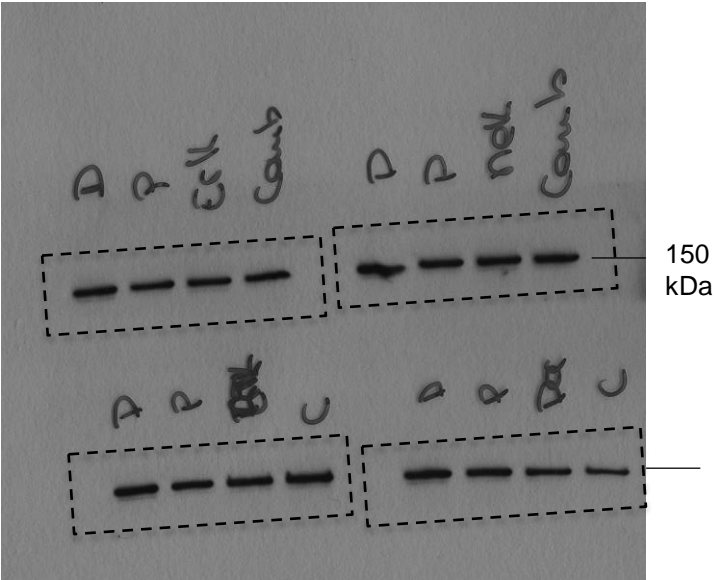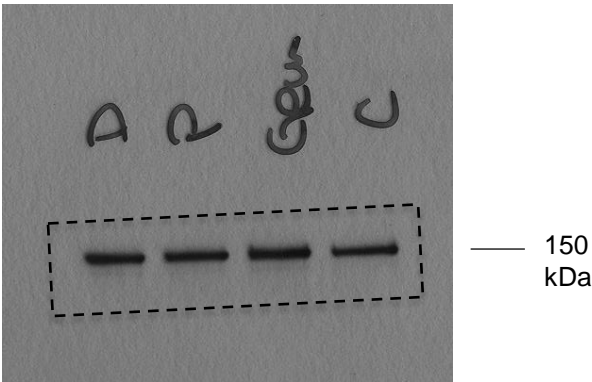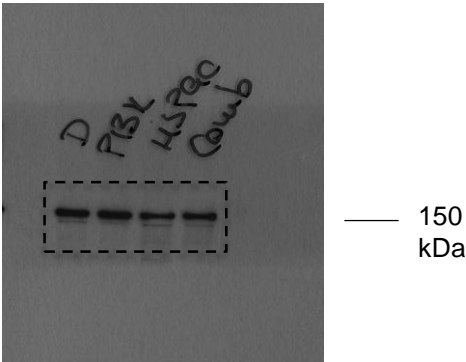

TRF1

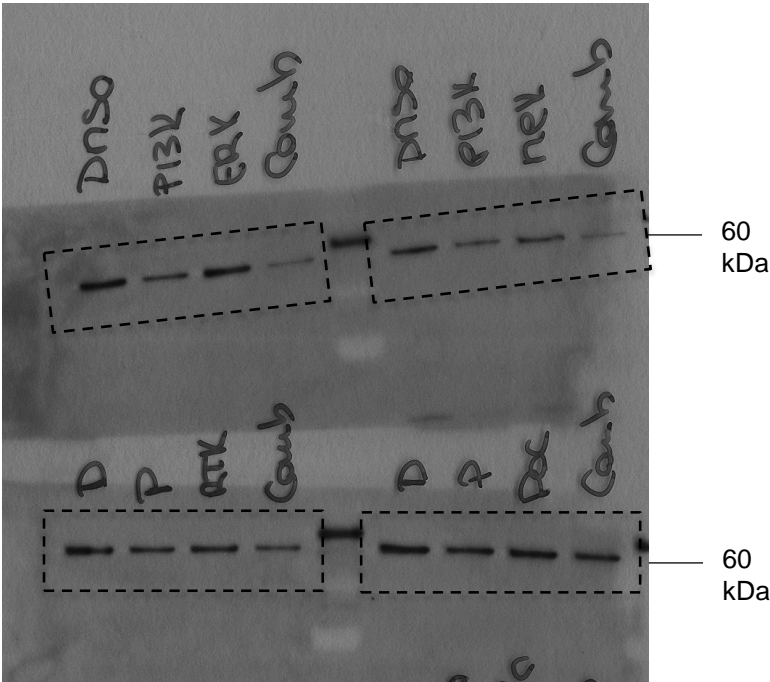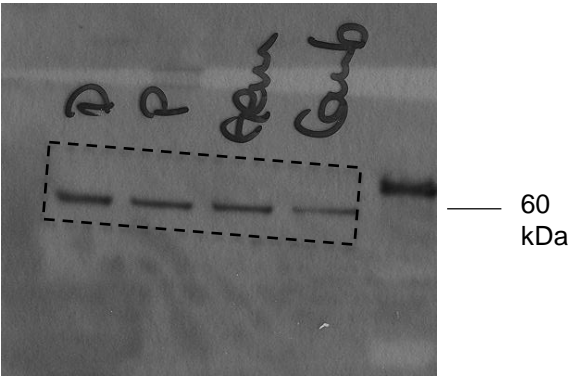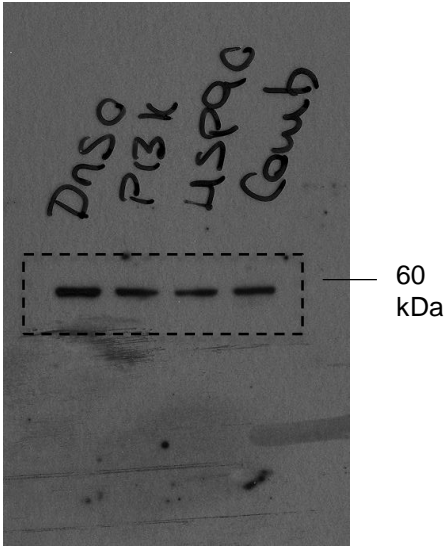

Supplement: Supplementary file 9 — Source Data for Figure 7 [file EMMM-11-e10292-s007.pdf]
